# A machine learning correction for DFT non-covalent interactions based on the S22, S66 and X40 benchmark databases

**DOI:** 10.1186/s13321-016-0133-7

**Published:** 2016-05-03

**Authors:** Ting Gao, Hongzhi Li, Wenze Li, Lin Li, Chao Fang, Hui Li, LiHong Hu, Yinghua Lu, Zhong-Min Su

**Affiliations:** School of Computer Science and Information Technology, Northeast Normal University, Changchun, 130117 China; Institute of Functional Material Chemistry, Faculty of Chemistry, Northeast Normal University, Changchun, 130024 China

**Keywords:** Non-covalent interactions, Density functional theory, Machine learning correction, Computational accuracy, Feature selection

## Abstract

**Background:**

Non-covalent interactions (NCIs) play critical roles in supramolecular chemistries; however, they are difficult to measure. Currently, reliable computational methods are being pursued to meet this challenge, but the accuracy of calculations based on low levels of theory is not satisfactory and calculations based on high levels of theory are often too costly. Accordingly, to reduce the cost and increase the accuracy of low-level theoretical calculations to describe NCIs, an efficient approach is proposed to correct NCI calculations based on the benchmark databases S22, S66 and X40 (Hobza in Acc Chem Rev 45: 663–672, 2012; Řezáč et al. in J Chem Theory Comput 8:4285, 2012).

**Results:**

A novel type of NCI correction is presented for density functional theory (DFT) methods. In this approach, the general regression neural network machine learning method is used to perform the correction for DFT methods on the basis of DFT calculations. Various DFT methods, including M06-2X, B3LYP, B3LYP-D3, PBE, PBE-D3 and ωB97XD, with two small basis sets (i.e., 6-31G* and 6-31+G*) were investigated. Moreover, the conductor-like polarizable continuum model with two types of solvents (i.e., water and pentylamine, which mimics a protein environment with ε = 4.2) were considered in the DFT calculations. With the correction, the root mean square errors of all DFT calculations were improved by at least 70 %. Relative to CCSD(T)/CBS benchmark values (used as experimental NCI values because of its high accuracy), the mean absolute error of the best result was 0.33 kcal/mol, which is comparable to high-level ab initio methods or DFT methods with fairly large basis sets. Notably, this level of accuracy is achieved within a fraction of the time required by other methods. For all of the correction models based on various DFT approaches, the validation parameters according to OECD principles (i.e., the correlation coefficient *R*^2^, the predictive squared correlation coefficient *q*^2^ and $q_{cv}^{2}$ from cross-validation) were >0.92, which suggests that the correction model has good stability, robustness and predictive power.

**Conclusions:**

The correction can be added following DFT calculations. With the obtained molecular descriptors, the NCIs produced by DFT methods can be improved to achieve high-level accuracy. Moreover, only one parameter is introduced into the correction model, which makes it easily applicable. Overall, this work demonstrates that the correction model may be an alternative to the traditional means of correcting for NCIs.

**Graphical abstract:**
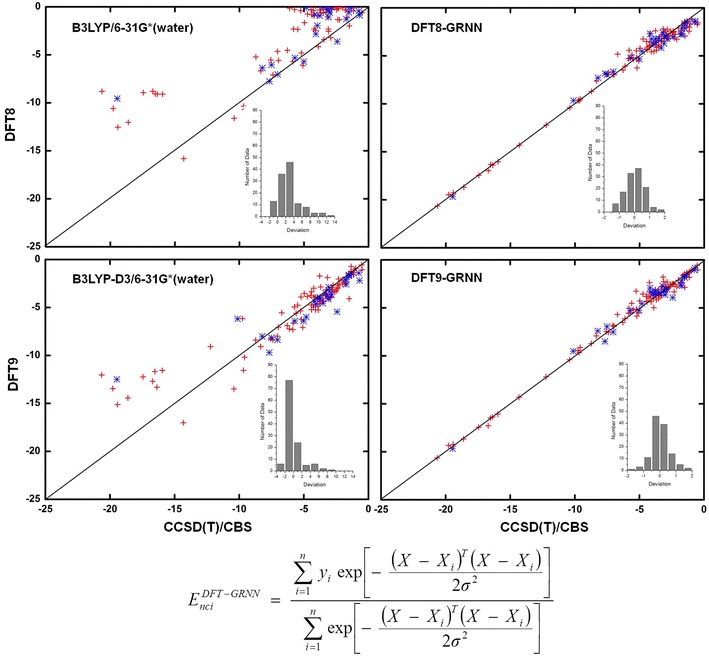
A machine learning correction model efficiently improved the accuracy of non-covalent interactions(NCIs) calculated by DFT methods. The application of the correction model is easy and flexible, so it may be an alternative correction means for NCIs by first-principle calculations.

**Electronic supplementary material:**

The online version of this article (doi:10.1186/s13321-016-0133-7) contains supplementary material, which is available to authorized users.

## Background

Non-covalent interactions (NCIs) are crucial in bio-molecular structures, supramolecules and various chemical reactions [1–4]. Because of the inherent intricacy of NCIs, their measurement is challenging, especially for complex biological systems. Therefore, computational methods are important tools for exploring NCIs. However, the accurate calculation of NCIs is quite demanding because such rigor requires coupled cluster or MPn levels of theory with large basis sets [e.g., complete basis set limit CBS, aug-cc-pVDZ, 6-311+G(3df, 2p)] [5]. The CCSD(T) method with a complete basis set description (i.e., CCSD(T)/CBS), which involves taking single and double electron excitations iteratively and triple electron excitation perturbatively, can provide a highly accurate description of various types of noncovalent complexes. Although this approach is considered to be the golden standard of computational methodologies, it is impractical for molecules with more than 100 atoms [6, 7]. Therefore, it is challenging to obtain accurate NCIs for medium- or large-sized molecules with reasonable computer resources. Compared with covalent bonds, NCIs are weak, highly susceptible to the environment and diversified. Generally, NCIs are classified into four categories: electrostatic (e.g., hydrogen bonding and ion-pairing), π-effect (e.g., cation–π, π–π stacking), van der Waals forces (e.g., dispersion attractions, dipole–dipole and dipole–induced dipole interactions) and hydrophobic. Among NCIs, the magnitude of hydrogen bonding is larger than that of most other NCIs, and hydrogen bonding combines electrostatic, polarization, exchange-repulsion, charge transfer, and even dispersion. Detailed energy decomposition analyses have shown that every interaction between two molecular systems involves a combination of multiple interactions that makes the interaction strong enough to maintain the stability of the molecular structures [6]. Although the magnitude of each NCI (i.e., several kilocalories) is much smaller than that of covalent bond interactions (i.e., hundreds of kilocalories), a dramatic effect may be observed in ligand binding, transition states, and biological systems [6]. Some special types of dispersion interactions, such as C–H···π, N–H···π, and halogen bonding, usually must be investigated individually [8, 9]. The significance of certain NCIs in biological systems remains largely uninvestigated [3, 8, 10]. These reports indicate that NCIs are intrinsically complicated and difficult to calculate with high accuracy.

Quantum chemical methods have become a routine tool for studying molecular systems. Density functional theory (DFT) methods are the most often used quantum chemical methods because of their low cost and satisfactory performance. However, DFT methods are deficient with respect to the calculation of NCIs. Recently, there has been significant effort to incorporate dispersion interactions in DFT methods, and great progress has been made [11–18]. However, further improvement in accuracy for NCI calculations is desirable. Regarding the forms of the dispersion corrections, in general, there are three types of NCI-corrected DFT methods. The parameterized NCI correction methods are standard hybrid DFT functionals with parameters optimized using training sets of benchmark interaction energies. Methods of this type include M05-2X and M06-2X [14], where the adjustable parameters have been fit to a ‘training set’ of molecules. The accuracy of such parameterized methods usually depends on the benchmark databases; for this reason, the accuracy of these methods may not be reliable for molecules that are not in the benchmark database. Dispersion correction methods, such as the DFT-D series, are flexible because the dispersion term can be added to any DFT method. Thus, the addition of correction terms can improve the calculation of NCIs [17]. However, dispersion interactions comprise only a fraction of the total NCIs. The long-range corrected hybrid density functionals, such as the ωB97 series [15, 16, 18], can be included to improve the performance when calculating NCI systems. However, these methods can only partly solve the accuracy of long-distance interactions. Although the results obtained with these corrected functionals are usually improved for most applications, there is no systematic way of improving them, and high accuracy by low levels of theory or for large molecules (i.e., >100 atoms) is difficult to achieve.

Machine learning methods have been implemented to process large data sets in many fields. In the past decade, machine learning methods have been successfully applied in the field of quantum chemistry to improve the accuracy of quantum chemical calculations for large molecules. In 2003, we applied neural networks to improve the accuracy of DFT calculations for the first time. In that paper, neural networks were used to correct the errors associated with B3LYP/6-311+G(d,p) calculations for the heats of formation $(\Delta H_{f}^{\theta } )$ of 180 organic molecules. The RMSE of the calculated $\Delta H_{f}^{\theta }$ were dramatically reduced from 21 to ~3 kcal/mol [19]. Thereafter, this strategy has been used to solve different types of accuracy problems for quantum chemical calculations, including absorption energies and Gibbs free energy [20–28]. In practical applications, the incorporation of quantum chemical methods and machine learning methods can be called a ‘GOLDEN’ combination because the advantages of both methods can be fully utilized; for example, machine learning methods can use the essential information captured by quantum chemical methods to reduce calculation errors caused by inherent approximations in the level of theory and limited basis sets. The essential feature of such a combination is to take the calculated properties of interest obtained by quantum chemical methods as the primary descriptor. Because the calculated values include all of the essential information of the property of interest, the systematic and random errors from various aspects of the calculations are easy to reduce. Thus, the accuracy of the quantum chemical calculations can be markedly improved, which enables low-level quantum chemical calculations to be performed with higher accuracy. Moreover, the use of machine learning methods is likely to uncover important factors that may affect the accuracy of the target properties. Therefore, this approach may reveal a new strategy for developing a correction term(s) for quantum chemical methods.

To improve the accuracy of DFT calculations for NCIs and investigate the factors that affect weak interactions, herein we propose a new correction for DFT NCI calculations through a combination of DFT and machine learning methods. In the following, the complex correction model is described according to the steps of model establishment. The model includes DFT calculations for the benchmark databases and the development of a stepwise machine learning correction model: data division, descriptor selection, regression and validation. Detailed discussions of the correction model and concluding remarks are presented following a description of the method.

## Methods

In recent years, a variety of means, dispersion corrections, long-range corrections and new parameterizations have been developed for DFT functionals to obtain reasonable descriptions of NCIs [14–18]. In this study, we propose a new simple form for the NCI correction for DFT methods. Specifically, a machine learning correction term can be used with many DFT functionals. This NCI correction is based on DFT calculations and a machine learning correction expressed as Eq. 1:

1 $$E_{nci}^{DFT - GRNN} = E_{nci}^{DFT} + E_{nci}^{Corr}$$

$E_{nci}^{DFT - GRNN}$ is the NCI after machine learning correction, $E_{nci}^{DFT}$ is the NCI calculated by the DFT methods and $E_{nci}^{Corr}$ is the correction that is improved by the machine learning method. With this approach, the correction is obtained by machine learning methods on the basis of the DFT calculations. This approach is an empirical method and the prediction model is established using DFT calculated NCIs as the primary descriptor; thus it is more efficient and more applicable than those that directly improve the DFT functionals. Plus, it also possesses good flexibility. Indeed, the trained correction term can be applied with most quantum chemical calculations. With the obtained molecular descriptors, the accuracy of the corresponding quantum chemical calculations can be improved to higher-level first-principles calculations. Moreover, its computational cost is very low and improvements in accuracy for low levels of theory are very likely because of the machine learning model capabilities. Furthermore, for the accuracy of the descriptor is not important for the machine learning calculations. The calculated descriptors are only required to reflect the qualitative trend of certain properties, which is easily achieved with quantum chemical methods with minimal basis sets. Therefore, small basis sets are sufficient for describing molecular systems, and the correction model can be readily applied to a wide range of molecules and various DFT methods as well as other first-principle methods. Notably, the method is not restricted to minima molecular geometries such that optimized structures with negative frequencies are also tolerable. Because this method is based on DFT calculations, the basic requirement of application is that a successful DFT (quantum chemical) calculation must be performed for the molecular descriptor calculation. To establish a general correction model for DFT methods and to show the flexibility of the model, we explored various DFT methods using this correction. A variety of functionals were chosen, including M06-2X, B3LYP, B3LYP-D3, PBE, PBE-D3 and ωB97XD. We note that the B3LYP and PBE functionals represent the DFT methods with or without fitting parameters, respectively.

### DFT calculations

DFT calculations were first performed to obtain quantum molecular descriptors. The benchmark databases of NCIs developed by Hobza et al. offer an excellent opportunity for novel computational techniques to examine NCIs [29–31]. In our calculations, three typical benchmark databases with equilibrium structures have been used (i.e., S22, S66 and X40). The three databases include various NCI complexes with important bonding motifs, H-bonded, dispersion-dominated, mixed, and halogen bonded complexes. The databases also cover a wide range of sizes and interaction strengths of NCI complexes. The initial geometries of the database molecules were taken from published supplementary materials [29–31]. The geometry optimizations and energy calculations were performed at the same level of theory. The downloaded structures in the references were not used here because the correction is meant to make predictions for molecules that are newly discovered or studied.

Regarding reference NCI values, the NCIs obtained by the CCSD(T)/CBS level of theory are taken as the target or reference experimental values of NCIs for building the correction models. The reason is that CCSD(T)/CBS is considered the golden standard of computational methodologies and its associated NCIs are highly accurate. By this means, two obstacles for a machine learning model can be solved: experimental NCIs and expansion of the database. Therefore, the correction model can be further improved by easily adding more molecules in the databases with accurate NCIs determined by the CCSD(T)/CBS level of theory. In addition, because the highly accurate NCIs determined herein by CCSD(T)/CBS are taken as experimental values, they are not considered calculated values under certain computational conditions any longer. Accordingly, the DFT calculations in this study are not confined to calculations in vacuum. We note that adopting gas phase experimental values as the targets for solution phase DFT calculations is not appropriate. Including a solvent model is like introducing a systematic error to the DFT calculations when comparing with gas phase experimental values. Fortunately, such protocols do not affect the performance of the machine learning correction models because the systematic errors can be easily removed, which is also one of the most important advantages in combining machine learning methods with quantum chemical calculations. That is, the calculations expose trends in the properties, which are possibly more important than the accuracy of the descriptors. The advantage allows us to perform either a gas phase or liquid phase descriptor calculation for an experimental target. In our previous works, we obtained the same correction accuracy using different input accuracies [19, 28]. This study also illustrates that input descriptors with different levels of accuracy were corrected to the same level of accuracy. The DFT methods M06-2X, ωB97XD, B3LYP, B3LYP-D3, PBE and PBE-D3 were used to calculate NCIs. For the M06-2X and ωB97XD methods, solvent effects have been considered using the conductor-like polarizable continuum model (C-PCM) with two types of solvents (i.e., water and pentylamine, which, with an epsilon value of 4.2, was chosen to mimic a protein’s environment). For the M06-2X method, the diffuse basis set effect was also investigated by comparing the results using the 6-31G* and 6-31+G* basis sets, which, although relatively small, make the model practical for large complexes. The M06-2X and ωB97XD calculations were performed using the Gaussian 09 program package [32]. However, this program has not implemented the 6-31G* and 6-31+G* basis sets for Bromine (Br) or Iodine (I). Thus, the polarization ECP basis set LANL2DZDP, which can be used for most metallic elements, was used for these atoms. B3LYP, B3LYP-D3, PBE and PBE-D3 calculations with the pure basis set 6-31G* were performed using the ORCA 3.0 quantum chemical program [33].

### Machine learning correction

The machine learning correction was constructed using a step-wise procedure: descriptor selections, data division, regression and validation. The detailed descriptions of each step are presented as follows. All values are normalized to [−1, 1] in the machine learning correction steps.

#### Data division

To maintain a balance between the training and test sets, the distance-dependent algorithm called, SPXY(sample set partitioning based on joint X–Y distance), a Ken-Stone improved method, is adopted [34, 35]. According to the joint x–y distances in Eq. 2, the training set and test set are partitioned such that the training set is concentrated in certain ranges or the maximal point is removed from the training set [34, 35].

2 $$d_{xy} (p,q) = \frac{{d_{x} (p,q)}}{{\max_{p,q \in [1,N]} d_{x} (p,q)}} + \frac{{d_{y} (p,q)}}{{\max_{p,q \in [1,N]} d_{y} (p,q)}};\quad p,q \in [1,N]$$

#### Partial least square (PLS) descriptor selection

Molecular descriptors represent the essential features of a molecule and can be considered its fingerprint. In a machine learning model, molecular descriptors can be the inputs of regression methods, and a quantitative structure activity/property relationship (QSA/PR) can be established between the inputs and output (targets/endpoints). Therefore, molecular descriptors markedly affect the quality of a regression model [36–38]. Usually, molecular descriptors can be obtained in various ways, including quantum chemical calculations, molecular mechanical calculations, and structure analyses. In our calculations, we sought to take full advantage of quantum chemical calculations while keeping the modeling as simple as possible. For this reason, only descriptors from quantum chemical calculations and constitutional descriptions of molecular structures were used to construct the model.

Screening of the molecular descriptors is an important step that is intended to avoid redundancy and noise of the extracted information. In this correction approach, PLS is used to select the most significant descriptors. PLS is a recently developed generalization of multiple linear regressions (MLR) and is a multivariate statistical data analysis method for modeling multiple variables. In addition to being a feature extraction method, it is also a regression model. This approach has become popular because it is capable of analyzing large amounts of data that are strongly correlated with noisy and large dimensional X-variables. It has also been found to be a very efficient data dimensionality reduction method [39]. Herein, PLS is used to screen the molecular descriptors; that is, the method selects the most significant descriptors from all of the available descriptors according to the PLS fitting coefficients.

#### GRNN regression modeling

**Fig. 1:**
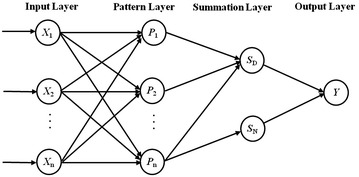
The structure of GRNN

The general regression neural network (GRNN) proposed by Specht [40] is a nonlinear regression method that is able to process data with high mapping capability within a flexible network. Notably, the GRNN method is robust when performing these calculations. The GRNN method shows a high learning rate and is asymptotic for the majority of samples. Moreover, its prediction is independent of the number of samples (i.e., the method is suitable for the regression of even a small number of samples). Compared with other machine learning methods, including genetic algorithm (GA), support vector machine (SVM) and back propagation neural networks (BPNN), GRNN can better reduce the training time while guaranteeing the quality of the regression model. The GRNN structure consists of four layers: input, pattern, summation and output layer (Fig. 1). The outputs are obtained by Eqs. 3–5.

3 $$p_{i} = \exp \left[ { - \frac{{(X - X_{i} )^{T} (X - X_{i} )}}{{2\sigma^{2} }}} \right],\quad i = 1,2, \ldots ,n$$

4 $$X = \left[ {x_{1} ,x_{2} , \ldots ,x_{n} } \right]^{T}$$

5 $$y = \frac{{S_{N} }}{{S_{D} }},\quad S_{D} = \sum\limits_{i = 1}^{n} {p_{i} } ,\quad S_{N} = \sum\limits_{i = 1}^{n} {y_{i} } p_{i}$$

where x_n_ is the neuron of the input layer, *p*_*i*_ is the neuron of the pattern layer such that the number of the pattern neuron is identical to the number of input samples, *X* is the transposed matrices of input neurons, *X*_*i*_ is the input neuron corresponding to the *i*th pattern neuron and σ is the smoothing factor that determines the shape of the function. Each pattern neuron corresponds to a training sample, and the Gaussian function is treated as the activation of the kernel function, which enhances the learning rate. *S*_*D*_ and *S*_*N*_ are the summation of the pattern neurons, *y* is the output and *y*_*i*_ is the experimental value of the training set.

To obtain a reliable and stable model, K-fold cross-validation is employed when training the network. In this approach, there are N samples in the training set, which are evenly divided into K groups. K − 1 groups are chosen as the training samples and the remaining sample is assigned as the validation sample. The network loops K times and the results of each cycle are compared. The best prediction accuracy of the input data sets is then selected to generate the GRNN model. The descriptor selection and regression modeling are fulfilled within the training set.

#### Model validation

To validate our models, we calculated validation parameters for our correction model according to the principles of the Organization for Economic Cooperation and Development (OECD) [41]. These parameters are the correlation coefficient *R*^2^, predictive squared correlation coefficient *q*^2^ and $q_{cv}^{2}$ obtained from cross-validation, mean absolute error (MAE) and root mean square error (RMSE), which represent the goodness-of-fit, robustness and predictive behavior of the model, respectively [42]. Generally, the fitting power in terms of *R*^2^ is larger than the stability power in terms of $q_{cv}^{2}$ (values of $q_{cv}^{2}$ and *q*^2^ larger than 0.5 are valid). If $R^{2} - q_{cv}^{2} > 0.3,$ then this may indicate that the established model is over-fit [43]. All of these parameters have been calculated to evaluate the correction models.

## Results and discussions

### Databases

In our correction model, the NCIs of the benchmark databases S22, S66 and X40 are examined. There are 125 different molecules in the benchmark databases. Of these, 121 molecular dimers were used in this study, whereas four molecules were discarded because of failures to optimize them with the chosen DFT methods. The molecules in the databases are classified into four types according to the dominant NCIs that are present: dispersion, hydrogen bonding, mixed complexes and halogen interactions. Various important NCI interaction motifs are included [29–31]. The numbers of NCI molecules in each class used in the correction model and mean values of the NCIs are listed in Table 1. Clearly, the mean NCIs of the H-bonded complexes are much lower than the other three types, which show the enhanced stability of H-bonded complexes relative to the other NCI dominated molecular systems.

**Table 1:** The mean values (kcal/mol) of CCSD(T)/CBS benchmark interactions and the number of four NCI-dominated molecular complexes

Types	Number	Mean
H-bonded complexes	29	−10.33
Dispersion complexes	30	−3.94
Mixed complexes	26	−3.70
Halogen complexes	36	−3.43

### NCI calculations with DFT methods

The geometries of molecules are important for the calculation of accurate DFT interaction energies. Therefore, the optimized structural profiles are kept identical to those found in the benchmark databases. In the X40 database for the molecules HX–MeOH (X = Cl, Br and I), the DFT methods overestimate the interaction between H and C on MeOH or underestimate the interaction between H and X. Accordingly, the optimized structures of these molecules cannot be obtained without constraints, and thus, these molecules were omitted from our study. The NCI energy is calculated by $E_{nci} = E_{AB} - (E_{A} + E_{B} ).$ Because NCI systems bound via weak interactions between the fragments are not as stable as covalently bonded systems, the global minimum sits in a shallow potential energy well. From our calculations, it is observed that when hydrogen bonds dominant the optimized structure, minor changes in the hydrogen bonds, such as a length or angle, lead to a change in the structure from a stable minimum to a saddle point structure with at least one negative frequency. Thus, it is challenging to locate the stationary point of some structures, and negative frequencies exist for some molecules. However, this outcome does not affect the results of the correction model when using machine learning methods to perform the correction (i.e., the correct physics is necessary rather than the accuracy of the descriptors). The overall results are show in Table 2. To simplify the expression, the DFT methods are named from DFT1 to DFT11 in Tables 2, 3 and 4. These results show that with respect to the benchmark NCIs by the CCSD(T)/CBS level of theory, the RMSE values of most DFT methods with functional corrections are <2 kcal/mol, which is apparently better than the methods in which dispersion corrections are not incorporated into the functionals (i.e., DFT8 and DFT10), where the RMSE values are larger than 3 kcal/mol. The RMSE values of different types of functional corrections (i.e., DFT1-7, DFT9 and DFT11) are similar. It should be noted that the MAEs and RMSEs of the liquid phase calculations probably include systematic errors induced by comparing solvent models with experimental gas phase values.

**Table 2:** The validation parameters of DFT and GRNN correction models (RMSE & MAE units: kcal/mol)

	RMSE		MAE		DFT		GRNN		
	DFT	GRNN	DFT	GRNN	q^2^	R^2^	q^2^	q_cv_^2^	R^2^
M062X/6-31G*^(vac)^ (DFT1)	1.66	0.50	1.34	0.35	0.87	0.98	0.98	0.95	0.99
M062X/6-31G*^a^ (DFT2)	1.79	0.52	1.13	0.37	0.85	0.93	0.98	0.92	0.99
M062X/6-31G*^b^ (DFT3)	*1.43*	*0.46*	*1.00*	0.34	0.90	0.96	0.98	0.93	0.99
M062X/6-31+G*^a^ (DFT4)	2.59	0.54	1.45	*0.33*	0.68	0.89	0.98	0.96	0.99
ωB97XD/6-31G*^(vac)^ (DFT5)	1.77	0.47	1.55	0.35	0.85	0.98	0.98	0.96	0.99
ωB97XD/6-31G*^a^ (DFT6)	1.74	0.54	1.22	0.38	0.86	0.93	0.98	0.93	0.99
ωB97XD/6-31G*^b^ (DFT7)	1.46	*0.46*	1.17	0.34	0.90	0.95	0.98	0.94	0.99
B3LYP/6-31G*^a^ (DFT8)	3.98	0.62	3.01	0.46	0.25	0.80	0.97	0.92	0.98
B3LYP-D3/6-31G*^a^ (DFT9)	1.89	0.56	1.18	0.40	0.83	0.93	0.98	0.92	0.99
PBE/6-31G*^a^ (DFT10)	3.15	0.62	2.33	0.46	0.53	0.83	0.97	0.92	0.98
PBE-D3/6-31G*^a^ (DFT11)	1.96	0.59	1.33	0.45	0.82	0.91	0.97	0.92	0.98

The best results are shown in italics

^vac^The calculations are performed in vacuum

^a^The solvent is set as water (ε = 78.35)

^b^The solvent is set as pentylamine (ε = 4.20), which possess a similar dielectric constant as the protein environment (ε ~4.0)

**Table 3:** The mean values of the NCIs in four types of complexes and the RMSEs of DFT calculations and GRNN corrections relative to the CCSD(T)/CBS benchmark NCIs (Unit: kcal/mol)

Methods	Mean (RMSE/RMSE1^c^)			
	H-bonded	Dispersion	Mixed	Halogen
CCSD(T)/CBS	−10.33 (−/−)	−3.94 (−/−)	−3.70 (−/−)	−3.43 (−/−)
M062X/6-31G^*(vac)^ (DFT1)	−12.23 (2.12/0.59)	−4.84 (1.11/0.45)	−4.86 (1.31/0.43)	−4.39 (1.85/0.53)
M062X/6-31G^*a^ (DFT2)	−9.23 (3.13/0.54)	−3.84 (1.01/0.47)	−*3.79* (*0.65*/0.53)	−3.81 (1.31/0.52)
M062X/6-31G^*b^ (DFT3)	−9.96 (2.37/0.37)	−*4.07* (*0.78*/0.47)	−4.05 (0.68/0.49)	−*3.82* (*1.22*/0.49)
M062X/6-31+G^*a^ (DFT4)	−7.24 (4.78/0.76)	−3.22 (1.25/0.45)	−2.80 (1.18/0.43)	−2.91 (1.33/*0.47*)
ωB97XD/6-31G^*(vac)^ (DFT5)	−12.46 (2.20/0.42)	−5.37 (1.52/*0.42*)	−5.20 (1.58/0.51)	−3.98 (1.72/0.53)
ωB97XD/6-31G^*a^ (DFT6)	−9.37 (2.88/0.50)	−4.30 (1.38/0.64)	−4.01 (0.74/0.41)	−3.43 (1.25/0.55)
ωB97XD/6-31G^*b^ (DFT7)	−*10.24* (*2.06/0.34*)	−4.61 (1.28/0.46)	−4.34 (0.86/*0.40*)	−3.53 (1.35/0.56)
B3LYP/6-31G^*a^ (DFT8)	−6.76 (5.10/0.57)	0.23 (5.06/0.64)	−0.85 (3.09/0.60)	−2.23 (2.04/0.65)
B3LYP-D3/6-31G^*a^ (DFT9)	−8.94 (3.30/0.58)	−3.88 (1.32/*0.42*)	−3.74 (0.67/0.66)	−4.16 (1.24/0.57)
PBE/6-31G^*a^ (DFT10)	−8.27 (3.94/0.59)	−0.68 (4.08/0.55)	−1.72 (2.27/0.79)	−3.22 (1.80/0.56)
PBE-D3/6-31G^*a^ (DFT11)	−9.75 (2.99/0.59)	−3.54 (1.76/0.57)	−3.74 (0.70/0.58)	−4.45 (1.66/0.60)

The best results are shown in italics

^vac^The calculations are performed in vacuum

^a^The solvent is set as water (ε = 78.35)

^b^The solvent is set as pentylamine (ε = 4.20), which possess a similar dielectric constant as the protein environment (ε ~4.0)

^c^The RMSE after the GRNN correction

**Table 4:** The RMSE (kcal/mol) of benchmark databases by DFT methods with respect to CCSD(T)/CBS benchmark interactions

Methods	RMSE					
	S22 (DFT)	S22 (GRNN)	S66 (DFT)	S66 (GRNN)	X40 (DFT)	X40 (GRNN)
M062X/6-31G^*(vac)^ (DFT1)	1.41	0.57	1.84	0.48	1.85	0.52
M062X/6-31G^*a^ (DFT2)	2.55	0.58	1.67	0.48	1.31	0.52
M062X/6-31G^*b^ (DFT3)	1.82	0.42	*1.37*	0.45	*1.22*	0.49
M062X/6-31+G^*a^ (DFT4)	3.99	0.83	2.45	0.43	1.33	*0.47*
ωB97XD/6-31G^*(vac)^ (DFT5)	1.68	0.30	1.46	0.43	1.71	0.53
ωB97XD/6-31G^*a^ (DFT6)	2.36	0.60	1.70	0.50	1.25	0.55
ωB97XD/6-31G^*b^ (DFT7)	*1.56*	*0.28*	1.46	*0.43*	1.35	0.56
B3LYP/6-31G^*a^ (DFT8)	5.97	0.47	3.90	0.63	2.04	0.65
B3LYP-D3/6-31G^*a^ (DFT9)	2.78	0.41	1.79	0.59	1.24	0.57
PBE/6-31G^*a^ (DFT10)	4.66	0.57	3.07	0.67	1.80	0.56
PBE-D3/6-31G^*a^ (DFT11)	2.68	0.45	1.79	0.61	1.66	0.60

The best results are shown in italics

^vac^The calculations are performed in vacuum

^a^The solvent is set as water (ε = 78.35)

^b^The solvent is set as pentylamine (ε = 4.20), which possess a similar dielectric constant as the protein environment (ε ~4.0)

#### Diffuse basis function

We note that DFT4 performs worse than DFT1–3, even though it uses a slightly larger basis set. In contrast to 6-31G*, the diffuse function present in 6-31+G* is intended to improve the calculation of NCIs. Diffuse functions play an important role in NCI calculations because they account for the distant electronic density of an atom. However, in this calculation, the diffuse function instead shows negative effects without any advantages. Indeed, the RMSE of DFT4 is 2.59 kcal/mol, which is 0.8 kcal/mol larger than that of DFT2. To clarify the role of the basis functions, ωB97XD/6-31+G* was also performed for both the S22 and S66 databases; notably, similar results were obtained, with RMSEs for M06-2X/6-31+G* and ωB97XD/6-31+G* of 2.86 and 2.91 kcal/mol, respectively. These results indicate that the use of one diffuse function does not benefit the calculation of NCIs for this basis set in solvents. Because the means of the correction for these two DFT methods are different, the reason for this basis set effect remains unclear. Further studies on basis set effects are ongoing.

#### Solvation effects

Because NCIs usually occur in aqueous biological systems, our calculations were mainly performed with a description for a solvent. Two solvent environments were considered: water and protein. The protein environment (with a dielectric constant ε of ~4.0) [44] is mimicked by pentylamine (ε = 4.2). In vacuum, DFT1 and DFT5 provide good results. In solvent, the results show that the NCI values are more accurate in the protein environment than in water. As shown in Table 2, the NCIs calculated by DFT3 and DFT7 possess the smallest RMSEs (<1.5 kcal/mol) among all of the DFT methods examined. This result indicates that solvent effects play a crucial role in NCI systems and the choice of solvent may impact the accuracy of the calculations. Our calculations demonstrate that an environment that accounts for the protein is recommended for NCI calculations in the corresponding level of theory, and the proper choice of a dielectric value can improve the accuracy of the DFT calculations [45].

#### Dominant interactions in the complexes

The mean values of the NCI references, the DFT calculations and the RMSE and MAE of the NCIs for four types of complexes calculated by various DFT methods are listed in Table 3. A comparison of the mean NCI values of the four types of dominant interaction complexes indicates that the mean NCI values of hydrogen bonding complexes are larger than the other three types of complexes because of its strong hydrogen bonding interaction, which provides greater stabilization to the complexes than the other NCIs. In the gas phase calculations (i.e., DFT1 and DFT5), all of the average interactions are larger than the reference values, which indicate that all of the interactions are overestimated. In the solvent phase, screening effects in water are very strong, and thus the results underestimate the hydrogen bonded and dispersion complexes. However, when the dielectric constant is decreased to 4.2 (i.e., the protein environment), the DFT calculations are clearly improved, with the best results obtained with DFT3 and DFT7. In the solvent phase, the NCIs of the hydrogen-bonded complexes are all underestimated by the DFT methods. The best estimate for the H-bonded complexes is obtained by DFT7 (i.e., ωB97XD/6-31G* with a protein environment). Except for the H-bonded complexes, estimates of the NCIs do not follow a consistent trend. The best results are obtained by DFT3 (i.e., M062X/6-31G* with a protein environment) for dispersion and halogen interactions and DFT2 (i.e., M062X/6-31G* with water) for mixed complexes. Comparing DFT methods with dispersion corrections, most of the RMSEs for the entire dataset fall between 20 and 50 % of the mean NCI values. Without the dispersion correction, DFT8 (i.e., B3LYP/6-31G* with water) and DFT10 (i.e., PBE/6-31G* with water) do not give reasonable results, although the latter method is better than the former for the four types of complexes.

#### Molecules in the benchmark databases

The RMSEs of the DFT methods and the GRNN corrections for the databases are listed in Table 4. The RMSEs of the best DFT calculations for S22, S66 and X40 are 1.56 kcal/mol by DFT7 and 1.37 and 1.22 kcal/mol by DFT3 (highlighted in italics), respectively. These results indicate the best performance of the DFT methods for the three databases are achieved by DFT3 and DFT7. Comparing these results in terms of solvents, a protein environment is more appropriate for estimating NCIs and yields better results in the corresponding level of theory. Based on the overall performance, we suggest that DFT3 and DFT7 are suitable for economic calculations for medium- and large-size systems.

### GRNN correction model

#### Descriptor analyses

With the SPXY method, the entire database was divided into a training set (consisting of 91 molecules) and a test set (consisting of 30 molecules). Because the trends of various DFT-calculated NCIs are similar in terms of their correlation coefficients, we performed screening for only descriptors obtained by the M06-2X method. In the M06-2X calculations, the effects of both the basis set and solvents are considered. In total, there are 43 descriptors extracted from the quantum chemical calculations and constitutional descriptors, which are listed in Additional file 1: Table S1.

**Fig. 2:**
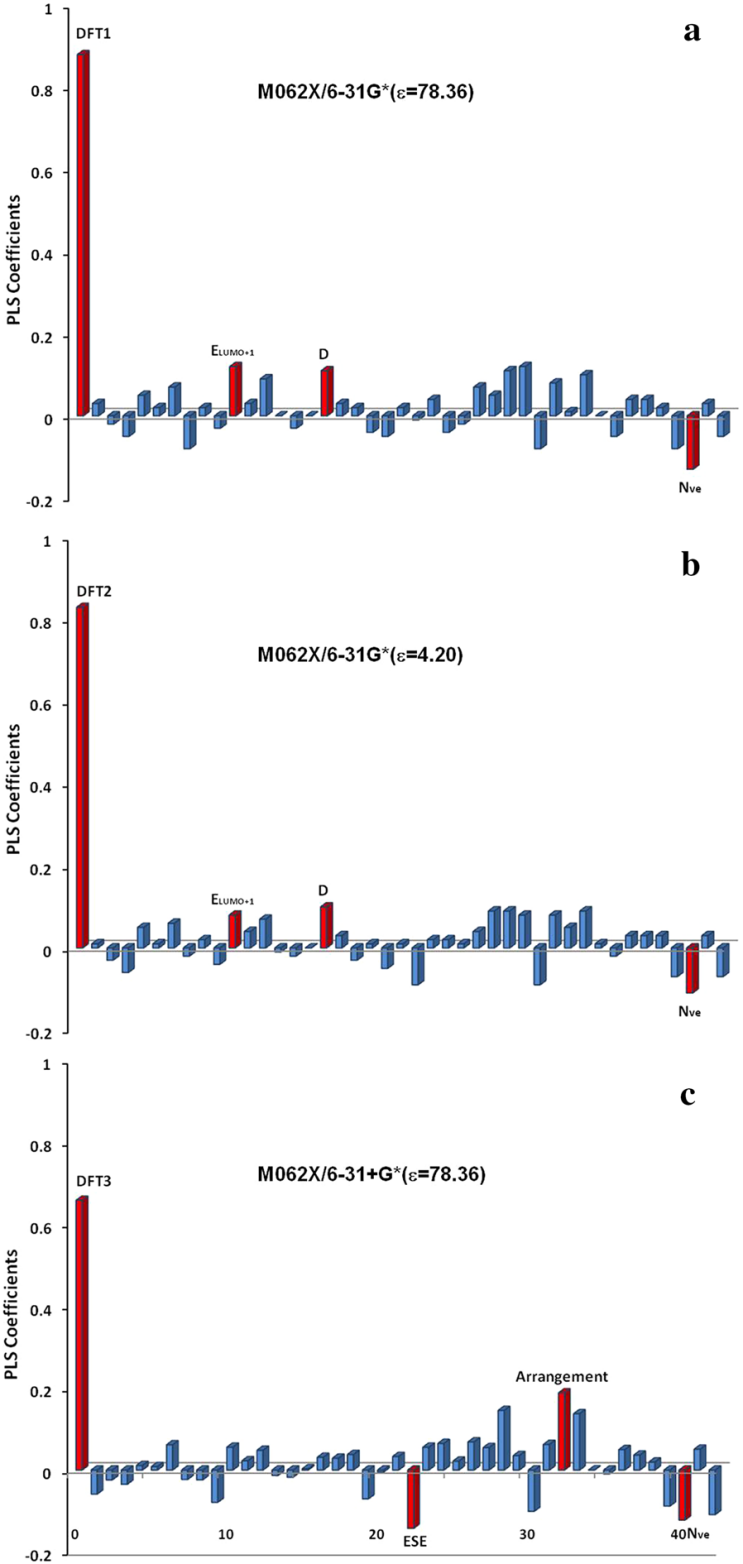
PLS coefficients for all molecular descriptors. The *red columns* are the selected descriptors from the calculations **a** M06-2X/6-31G* (water), **b** M06-2X/6-31G* (pentylamine), **c** M06-2X/6-31+G* (water) for the GRNN correction model, respectively

From the DFT calculations and structural analyses, of the forty-three molecular descriptors, 25 are quantum chemical descriptors, whereas the remaining are molecular structural descriptors, such as the number of atoms and the number of valence electrons. The results from the molecular descriptor screening using the PLS method are shown in Fig. 2 and Additional file 1: Table S1. In the PLS screening, the top 10 descriptors in terms of the PLS coefficients were chosen for further regression modeling. After optimizing the neural network, the optimal network consisted of four input descriptors.

In Fig. 2, the red columns are the selected descriptors. For M06-2X/6-31G* either in water or the protein environment, DFT-calculated NCIs (DFT1-2), the number of valence electrons (N_ve_), dipole moments (D), and the energy of the second lower unoccupied molecular orbital (E_LUMO+1_) are selected. For M06-2X/6-31+G* with water, four selected descriptors for regression calculations are shown in Fig. 2c (DFT3, N_ve_, ESE, Arrangement). However, two selected descriptors [i.e., the arrangement of monomers (Arrangement) and the electronic spatial extent (ESE)] are different from those used for the M06-2X/6-31G* methods. This difference may be ascribed to numerical differences of the descriptors from different basis set calculations. Thus, for M06-2X/6-31G* with different solvents, the selected descriptors are identical. Because the same basis set gives similar accuracy, the same selected descriptors for two basis sets with other DFT methods are adopted for the regression model.

**Fig. 3:**
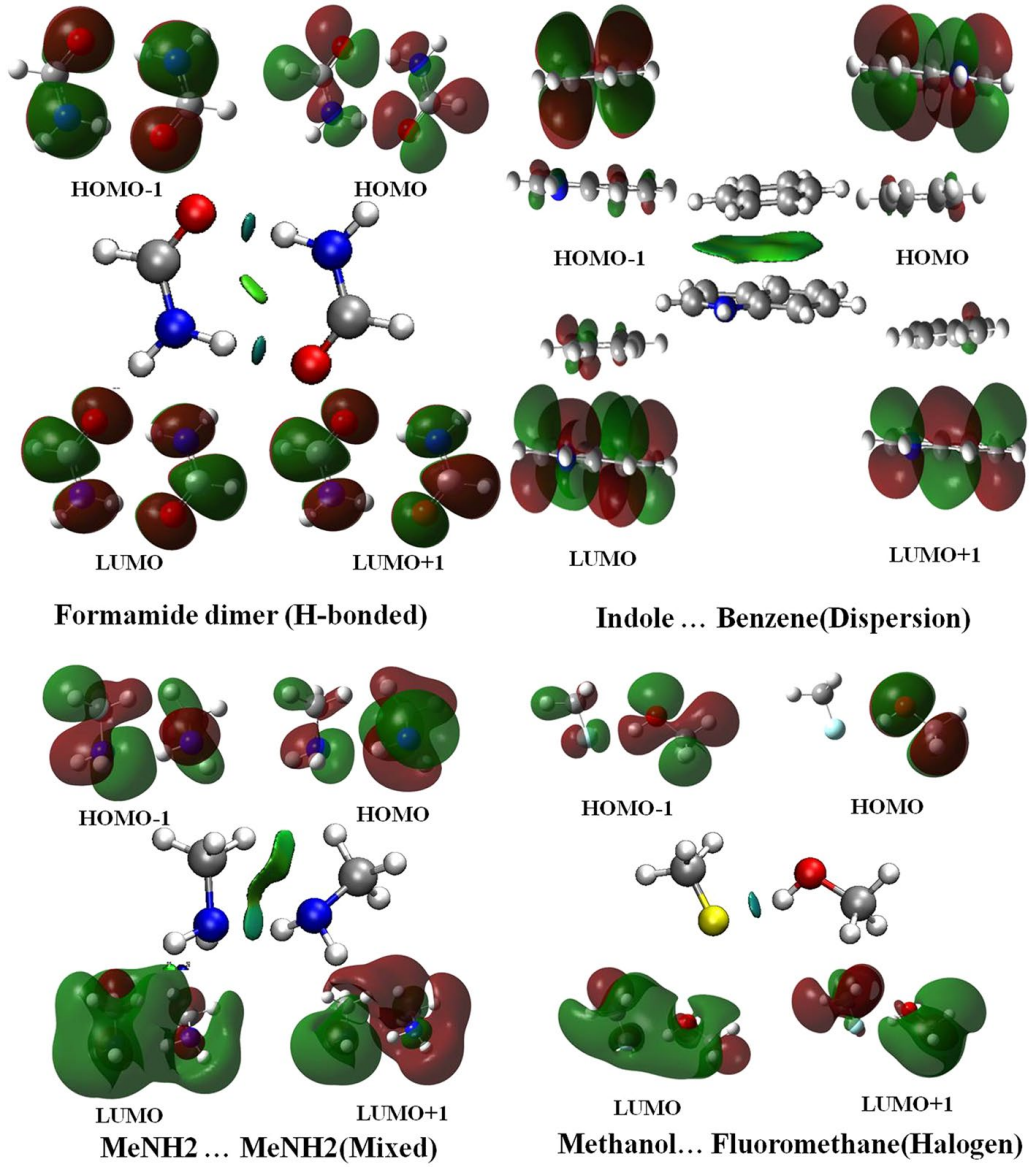
The NCI plots and electron density of the frontier molecular orbitals (Carbon: *grey*, Nitrogen: *blue*, Oxygen: *red*, Hydrogen: *white*, Chlorine: *yellow*)

The coefficients of the primary descriptor, DFT1-3, are much larger than the other selected descriptors (Fig. 2). The other descriptors show a similar significance and are used to amend the offset of the DFT NCI values. N_ve_ was selected by both basis set calculations, which may indicate that the nature of the NCIs is mainly electronic. Dipole–dipole interactions are very important for NCIs [46, 47], which may affect charge transfer, electron transition and excited state properties. Thus, there is little doubt that dipoles are also important for NCIs. The arrangement of the monomers in the NCI systems can influence the size of the interaction area between the monomers. Therefore, Arrangement is closely related to the magnitude of the interactions. The E_LUMO+1_ reflects the electronic properties of bonding, and ESE is the electron density distribution that indicates the molecular interaction space. The NCIs and the electron density of the frontier molecular orbitals of representative molecules from four types of complexes are plotted in Fig. 3. Although there is no loss or gain in the NCIs, the electron density of each monomer changes because of the interaction between monomers. However, from the frontier orbital distributions and the NCI plots [48], we cannot determine how the LUMO + 1 contributes to the dominant interactions. Thus, this result may be a numerical coincidence because the values of the PLS coefficients are quite close to each other (<0.2) for all of the descriptors except for the DFT NCI values. In total, there are two constitutional and four quantum chemical descriptors in the selected descriptors. It is not surprising that quantum chemical descriptors are more favorable than constituent descriptors because they possess more detailed information of the target property.

#### GRNN correction

For GRNN, the network structure and connection weights between neurons are determined if the study samples are assigned. In the GRNN modeling, there is only one parameter that requires optimization: the smoothing factor, σ. The regression value of σ determines the generalizability of the network; when σ approaches zero, the output becomes very similar to the objective value, although the predictive ability might be poor. In practice, network training is the process of optimizing the smoothing parameter. It is necessary to select reasonable smoothing parameters. Smaller smooth parameters (σ) give rise to stronger network approximation processes. Similarly, higher values of the smoothing parameter result in a smoother network approximation process but will increase the validation error. In this study, we used a loop test to determine the smoothing parameter. During training, the range of σ values was set as [0.1, 2] with a step size of 0.1. The optimal GRNN model was constructed using the σ value with the smallest validation errors. Through training, the best smoothing parameters (σ) were obtained for the 6-31G* (0.2) and 6-31+G* (0.1) basis sets. Notably, σ is insensitive to the DFT method, but appears to be influenced by the basis sets that are employed. Although a GRNN model was trained for each DFT method, only two σ values (i.e., 0.2 and 0.1) were obtained for the 6-31G* and 6-31+G* basis sets.

By GRNN regression, the functional form of the correction can be expressed as:

6 $$E_{nci}^{DFT - GRNN} = \frac{{\sum\nolimits_{i = 1}^{n} {y_{i} \exp \left[ { - \frac{{(X - X_{i} )^{T} (X - X_{i} )}}{{2\sigma^{2} }}} \right]} }}{{\sum\nolimits_{i = 1}^{n} {\exp \left[ { - \frac{{(X - X_{i} )^{T} (X - X_{i} )}}{{2\sigma^{2} }}} \right]} }}$$

As shown in the GRNN method section, *y*_*i*_ denotes the benchmark NCI of the training set, *X* is the transposed matrices of input neurons (four selected descriptors) and *X*_*i*_ is the input neuron corresponding to the *i*th pattern neuron, all of which are known inputs. Thus, in the correction model, there is an empirical parameter σ (0.2 and 0.1 for the 6-31G* and 6-31+G* basis sets, respectively) that is determined when the regression model is constructed. This correction term is easy to implement in quantum chemical programs, where it can be added as a subroutine after the quantum chemical calculations. Thus, using a low-cost DFT calculation to obtain molecular descriptors, NCIs by higher levels of theory can be achieved. Further, this approach could be applicable to a wider range of molecular systems than could be determined directly using higher levels of theory. The proposed correction model was generated in Matlab R2014b [49] with the GRNN code [50]. The Matlab code used for our correction model in Ref. [50] is presented in the Additional file 2.

The correction is obtained with this regression model. The overall corrected results presented in Table 2 show that the correction model improved all of the DFT results, irrespective of whether an inherent NCI correction was already present in the DFT functional. The GRNN correction RMSEs relative to the CCSD(T)/CBS benchmark NCIs were reduced from 1.43–3.98 to 0.46–0.62 kcal/mol, and the MAEs decreased from 1.00–3.01 to 0.33–0.46 kcal/mol. The best correction results were achieved by combinations of GRNN with both DFT3 and DFT7, which are based on the best DFT results. The correction for the methods without NCI corrections was significant; indeed, the RMSEs of DFT8 and DFT10 were reduced to 0.62 from 3.98 to 3.15 kcal/mol, respectively. All of the evaluation parameters of the GRNN model are larger than 0.92 (Table 2), indicating that the GRNN correction model is robust and possesses good predictability in terms of the principles outlined by the OECD. As shown in Table 3, for the different types of NCI-dominated complexes, the GRNN correction remarkably reduces the errors in various NCI systems. The best performance for each type of complex was 0.34 kcal/mol for H-bonded, 0.42 kcal/mol for dispersion, 0.40 kcal/mol for mixed and 0.47 kcal/mol for halogen complexes. Regarding the databases, the GRNN correction also performs well and is very stable (Table 4). The best performance for S22(0.28) and S66(0.43) on the basis of DFT7(ωB97XD/6-31G*) was similar to the results calculated with spin-component scaled MP2 SCS-MI-MP2/cc-PVTZ S22 (0.26 kcal/mol) and S66 (0.38 kcal/mol) reported in ref. 6, whereas the DFT methods used in this study required significantly less computational time than the MP2 methods.

**Fig. 4:**
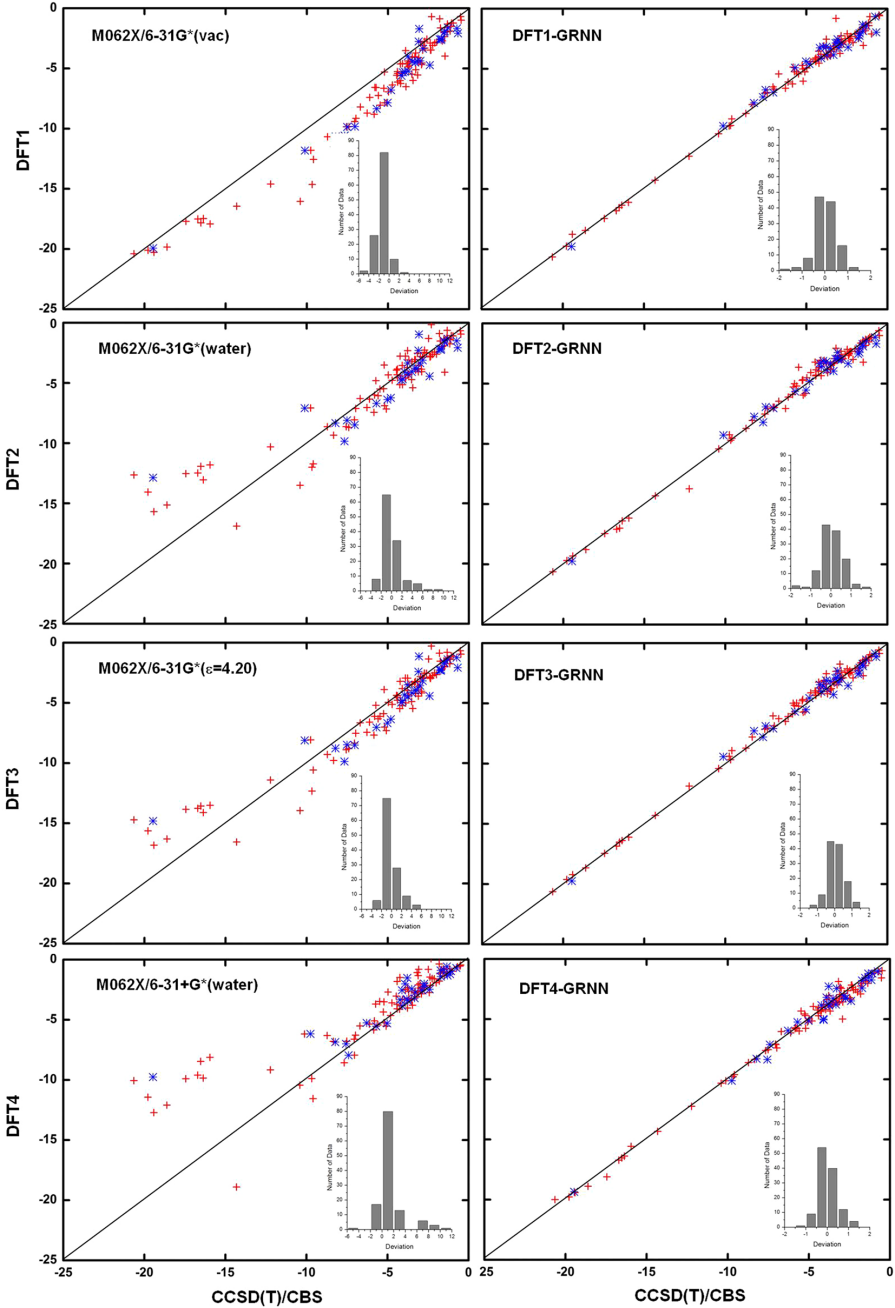
NCIs calculated by DFT M06-2X (*left*) and DFT-GRNN (*right*) versus benchmark values. The *insets* are the deviation (calculated-benchmark) distribution relative to the benchmark values in each calculation (training set: *red*; test set: *blue*)

**Fig. 5:**
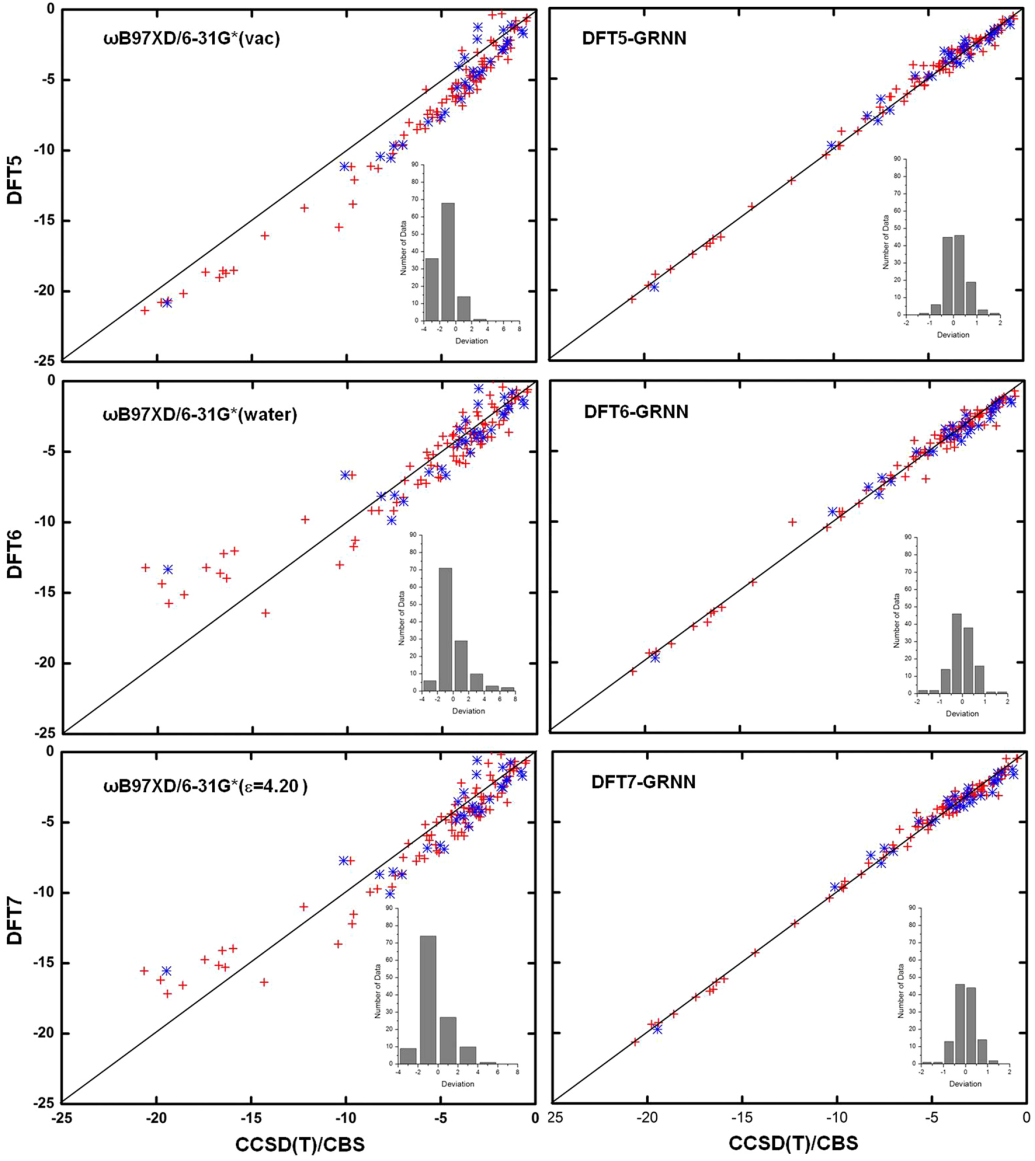
NCIs calculated by DFT ωB97XD and DFT-GRNN versus benchmark values. The *insets* are the deviation distribution relative to the benchmark values in each calculation (training set: *red*; test set: *blue*)

**Fig. 6:**
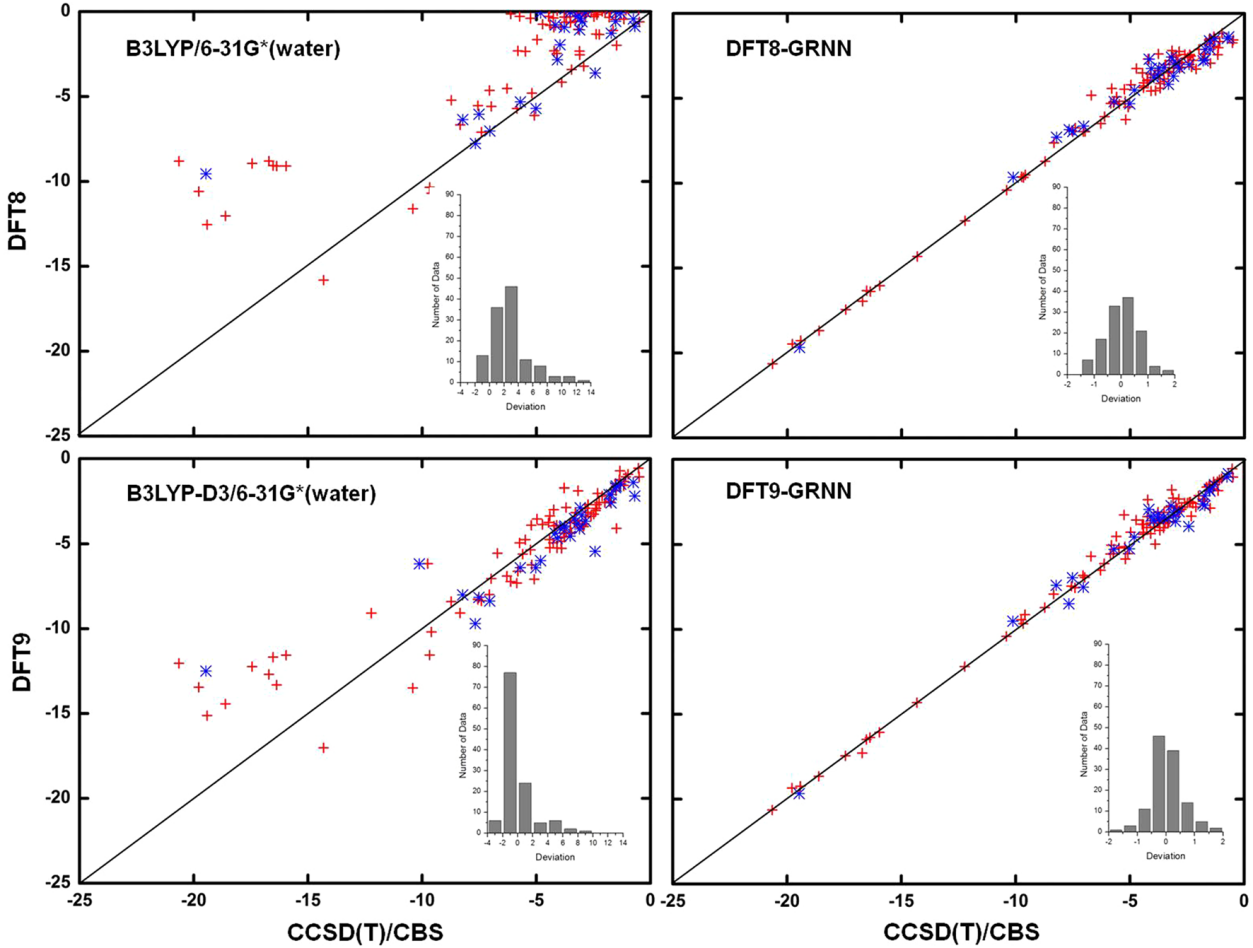
NCIs calculated by DFT B3LYP, B3LYP-D3 and DFT-GRNN versus benchmark values. The *insets* are the deviation distribution relative to the benchmark values in each calculation (training set: *red*; test set: *blue*)

**Fig. 7:**
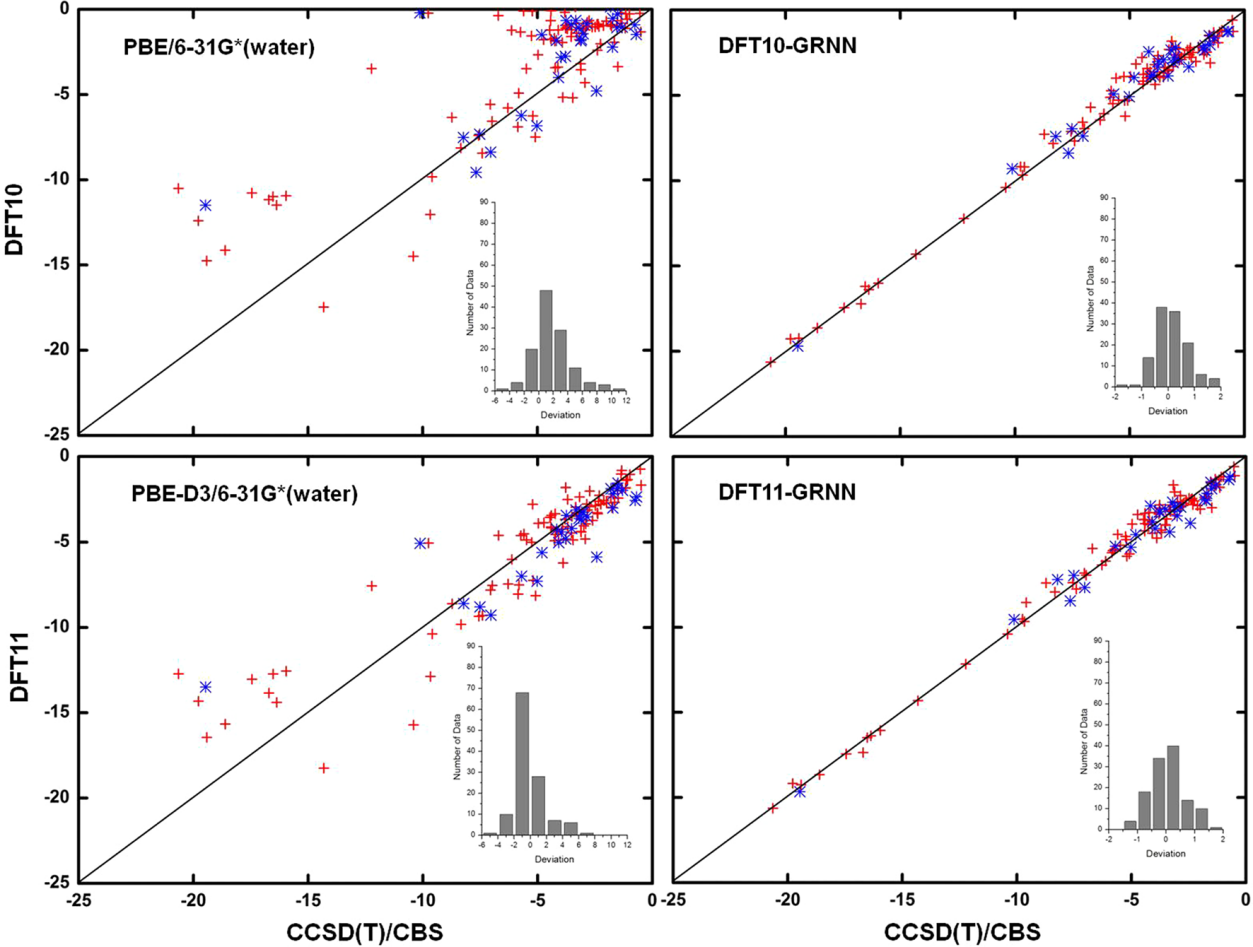
NCIs calculated by DFT PBE, PBE-D3 and DFT-GRNN versus benchmark values. The *insets* are the deviation distribution relative to the benchmark values in each calculation (training set: *red*; test set: *blue*)

The NCI results before and after the GRNN correction are plotted in Figs. 4, 5, 6 and 7 and Additional file 3: Table S2, Additional file 4: Table S3, Additional file 5: Table S4, Additional file 6: Table S5, Additional file 7: Table S6, Additional file 8: Table S7, Additional file 9: Table S8, Additional file 10: Table S9, Additional file 11: Table S10, Additional file 12: Table S11 and Additional file 13: Table S12 for M06-2X, ωB97XD, B3LYP, B3LYP-D3, PBE and PBE-D3, respectively. These figures show the calculations by DFT and the GRNN correction versus the CCSD(T)/CBS benchmark NCI values. Clearly, all of the NCI values are improved and move towards the CCSD(T)/CBS results after the GRNN correction is applied. All of the deviations (NCI_DFT_–NCI_GRNN_) of the DFT(1–11) calculations are reduced, and the range of error distributions is narrowed from [−3, 13] to [−2, 2] kcal/mol. The systematic errors are eliminated, and most of the values are close to zero. Moreover, the results with the correction included have no serious outliers, and the accuracy of the absolute values of most data is within chemical accuracy. As shown in the left panels of Figs. 6 and 7, the improvement of the DFT NCI calculations by adding the dispersion correction demonstrates the significance of the dispersion correction term in DFT functionals. After adding the dispersion (D3) correction, the errors of B3LYP and PBE are decreased and their accuracy is similar to that of other dispersion corrected DFT methods. The results after the GRNN correction is applied are similar, indicating that GRNNs are capable of correcting the results of DFT methods with no inherent NCI correction.

## Conclusions

A general NCI correction constructed by GRNN for DFT methods is proposed. NCI calculations have been a serious problem for DFT methods. Recent developments have improved these methods, although NCI calculations with DFT methods with small basis sets continue to possess large deviations. In this present work, a GRNN correction for DFT NCI calculations was applied to various DFT methods with or without inherent NCI corrections in the functional. The results show that with this new correction, all of the RMSEs of the tested DFT methods can be improved to chemical accuracy (i.e., 0.4–0.6 kcal/mol). Because the approach combines the strengths of both DFT and GRNN methods, it simultaneously achieves both efficiency and accuracy at a very low cost. The best accuracy obtained by GRNN based on ωB97XD/6-31G* is comparable with previous SCS-MI-MP2/cc-PVTZ calculations. In summary, the great advantages of this method are the following: (1) the efficiency and accuracy of the method is high, and it can be applied to large molecules with accuracy comparable to higher levels of theory; (2) the correction model does not strictly require accurate descriptors as inputs, provided that they correlate with properties in certain trends; and (3) in the GRNN correction, there is only one parameter that must be fit, and the obtained model is easy to implement in quantum chemical programs. Using the NCIs calculated by the CCSD(T)/CBS method as the target or reference experimental values for the correction model not only avoids the difficulty of finding experimental NCIs but also sets the stage for further improvements to the correction model by adding more molecules to the database using the results from CCSD(T)/CBS calculations. Moreover, the proposed state-of-the-art correction can be an alternative means for extending DFT methods to large systems with comparably high accuracy. Furthermore, we believe that this approach can also improve the accuracy of NCIs for other first-principle methods.

## Additional files

10.1186/s13321-016-0133-7 The full list of the molecular descriptors.
10.1186/s13321-016-0133-7 The Matlab code for GRNN corredction models.
10.1186/s13321-016-0133-7 The NCI, descriptors and errors based on M062X/6-31G*(vac).
10.1186/s13321-016-0133-7 The NCI, descriptors and errors based on M062X/6-31G* calculations.
10.1186/s13321-016-0133-7 The NCI, descriptors and errors based on M062X/6-31G*(ε=4.20) calculations.
10.1186/s13321-016-0133-7 The NCI, descriptors and errors based on M062X/6-31+G* calculations.
10.1186/s13321-016-0133-7 The NCI descriptors and errors based on WB97XD/6-31G*(vac) calculations.
10.1186/s13321-016-0133-7 The NCI, descriptors and errors based on WB97XD6-31G* calculations.
10.1186/s13321-016-0133-7 The NCI, descriptors and errors based on WB97XD/6-31G* (ε=4.20) calculations.
10.1186/s13321-016-0133-7 The NCI, descriptors and errors based on B3LYP/6-31G* calculations.
10.1186/s13321-016-0133-7 The NCI, descriptors and errors based on B3LYP-D3/6-31G* calculations.
10.1186/s13321-016-0133-7 The NCI, descriptors and errors based on PBE/6-31G* calculations.
10.1186/s13321-016-0133-7 The NCI, descriptors and errors based on PBE-D3/6-31G* calculations.
