# Supplementary material for: A machine learning correction for DFT non-covalent interactions based on the S22, S66 and X40 benchmark databases
Source: J Cheminform. 2016 May 3;8:24. doi: 10.1186/s13321-016-0133-7 (PMC4855356; doi:10.1186/s13321-016-0133-7)
Supplement: Supplementary file 1 — 10.1186/s13321-016-0133-7 The full list of the molecular descriptors. [file 13321_2016_133_MOESM1_ESM.docx]

**Table S1.The full list of the molecular descriptors**

| No. | **Symbols** | **Descriptors** | **PLS coefficients** | | |
| --- | --- | --- | --- | --- | --- |
|  |  |  | **DFT1** | **DFT2** | **DFT3** |
| **Quantum chemical descriptors (QD)** | | | | | |
| 1 | NCI | the non-covalent interactions calculated by DFT | 0.88 | 0.83 | 0.66 |
| 2. | Freq | the first frequency | 0.03 | 0.01 | -0.06 |
| 3 | E_d_ | the energy of molecular dimmer | -0.02 | -0.03 | -0.02 |
| 4 | E_a_ | the energy of A molecule ^a^ | -0.05 | -0.06 | -0.03 |
| 5 | E_b_ | the energy of B molecule ^a^ | 0.05 | 0.05 | 0.01 |
| 6 | P_D_ | the polarizability of molecular dimmer | 0.02 | 0.01 | 0.01 |
| 7 | ZPE | Zero point energy | 0.07 | 0.06 | 0.06 |
| 8 | E_HOMO-1_ | the energy of HOMO-1 ^b^ | -0.08 | -0.02 | -0.02 |
| 9 | E_HOMO_ | the energy of HOMO ^c^ | 0.02 | 0.02 | -0.02 |
| 10 | E_LUMO_ | the energy of LUMO ^d^ | -0.03 | -0.04 | -0.08 |
| 11 | E_LUMO+1_ | the energy of LUMO+1 ^e^ | 0.12 | 0.08 | 0.06 |
| 12 | g_HL_ | E_HOMO_-E_LUMO_ | 0.03 | 0.04 | 0.02 |
| 13 | P_A_ | the polarizability of A monomer | 0.09 | 0.07 | 0.05 |
| 14 | P_B_ | the polarizability of B monomer | 0.00 | -0.01 | -0.01 |
| 15 | Area | the area of molecular dimmer | -0.03 | -0.02 | -0.02 |
| 16 | V | the volume of molecular dimmer | 0.00 | 0.00 | 0.00 |
| 17 | D | the dipole of molecular dimmer | 0.11 | 0.10 | 0.03 |
| 18 | Q_A_ | the total charge of A monomer | 0.04 | 0.02 | 0.06 |
| 19 | Q_B_ | the total charge of B monomer | -0.04 | 0.02 | 0.06 |
| 20 | KE | kinetic energy | 0.03 | 0.03 | 0.03 |
| 21 | PE | potential energy | 0.02 | -0.03 | 0.04 |
| 22 | EE | electronic energy | -0.04 | 0.01 | -0.07 |
| 23 | NN | interactions among nucleuses | -0.05 | -0.05 | 0.00 |
| 24 | EN | interactions between electronic and nucleuses | 0.02 | 0.01 | 0.03 |
| 25 | ESE | electronic spatial extent | -0.01 | -0.09 | -0.14 |
| **Constituent descriptors (CD)** | | | | | |
| 26 | SP_3A_ | the number of sp3 hybridized carbon atoms in A | -0.02 | 0.01 | 0.02 |
| 27 | SP_2A_ | the number of sp2 hybridized carbon atoms in A | 0.07 | 0.04 | 0.07 |
| 28 | SP_A_ | the number of sp hybridized carbon atoms in A | 0.05 | 0.09 | 0.05 |
| 29 | SP_3B_ | the number of sp3 hybridized carbon atoms in B | 0.11 | 0.09 | 0.15 |
| 30 | SP_2B_ | the number of sp2 hybridized carbon atoms in B | 0.12 | 0.08 | 0.04 |
| 31 | SP_B_ | the number of sp hybridized carbon atoms in B | -0.08 | -0.09 | -0.10 |
| 32 | SP_3_/N_C_ | the proportion of sp3 hybridized carbon atoms among all the carbon atoms | 0.08 | 0.08 | 0.06 |
| 33 | Arrangement | the arrangement of molecular monomers | 0.01 | 0.05 | 0.19 |
| 34 | N_C_/N_A_ | the total number of hybridized carbon atoms/ NA | 0.10 | 0.09 | 0.14 |
| 35 | SP+SP2+/N_A_ | the proportion of sp and sp2 hybridized carbon atoms in molecules | 0.00 | 0.01 | 0.00 |
| 36 | Ca/Cb | the number of A carbon atoms/ the number of B carbon atoms | -0.05 | -0.02 | -0.01 |
| 37 | N_X_ | the number of halogen elements | 0.04 | 0.03 | 0.05 |
| 38 | χ | the electronegativity of halogen elements | 0.04 | 0.03 | 0.04 |
| 39 | N_A_ | the number of atoms | 0.02 | 0.03 | 0.02 |
| 40 | N_e_ | the number of total electrons | -0.08 | -0.07 | -0.09 |
| 41 | N_ve_ | the number of valence electrons | -0.13 | -0.11 | -0.12 |
| 42 | Freedom | the molecular degree of freedom | 0.03 | 0.03 | 0.05 |
| 43 | M_c_ | the distance between two mass centers | -0.05 | -0.07 | -0.11 |

^a^ One of the monomers of molecular dimmer is assigned to A and the other is B ^b^HOMO-1 is the second highest occupied molecular orbital.  ^c^HOMO is the highest occupied molecule orbital. ^d^LUMO is the lowest unoccupied molecular orbital. ^e^LUMO+1 is the second lowest unoccupied molecular orbital.
