# Supplementary material for: A machine learning correction for DFT non-covalent interactions based on the S22, S66 and X40 benchmark databases
Source: J Cheminform. 2016 May 3;8:24. doi: 10.1186/s13321-016-0133-7 (PMC4855356; doi:10.1186/s13321-016-0133-7)
Supplement: Supplementary file 2 — 10.1186/s13321-016-0133-7 The Matlab code for GRNN corredction models. [file 13321_2016_133_MOESM2_ESM.docx]

%%Matlab code for GRNN correction model

data1=xlsread('train'); %training set

data2=xlsread('test'); %test set

[row1,col1]=size(data1);

trainp=data1(:,2:col1); %The input of training set

[row2,col2]=size(data2);

testp=data2(:,2:col2); %The input of test set

traingoal=data1(:,1)';

testgoal=data2(:,1)';

p_train=trainp;

t_train=traingoal';

%% cross-validation

desired_spread=[];

mse_max=10e20;

desired_input=[];

desired_output=[];

result_perfp=[];

indices = crossvalind('Kfold',length(p_train),8);

k=1;

for i = 1:8

perfp=[];

disp(['the result of',num2str(i),'cross-validation'])

test1 = (indices == i); train = ~test1;

p_cv_train=p_train(train,:);

t_cv_train=t_train(train,:);

p_cv_test=p_train(test1,:);

t_cv_test=t_train(test1,:);

p_cv_train=p_cv_train';

t_cv_train=t_cv_train';

p_cv_test= p_cv_test';

t_cv_test= t_cv_test';

[p_cv_train,minp,maxp,t_cv_train,mint,maxt]=premnmx(p_cv_train,t_cv_train);

p_cv_test=tramnmx(p_cv_test,minp,maxp);

for spread=0.1:0.1:2;

net=newgrnn(p_cv_train,t_cv_train,spread);

% waitbar(k/80,h);

disp(['the value of spread is', num2str(spread)]);

test_Out=sim(net,p_cv_test);

test_Out=postmnmx(test_Out,mint,maxt);

error=t_cv_test-test_Out;

disp(['tne mse is',num2str(mse(error))])

perfp=[perfp mse(error)];

if mse(error)<mse_max

mse_max=mse(error);

desired_spread=spread;

desired_input=p_cv_train;

desired_output=t_cv_train;

end

k=k+1;

end

result_perfp(i,:)=perfp;

end;

disp(['the best value of spread',num2str(desired_spread)])

disp(['the best value of input'])

desired_input;

disp(['the best value of output'])

desired_output;

%%Using the best method to build GRNN network

net=newgrnn(desired_input,desired_output,desired_spread);

%%Using GRNN network to predict the test set

p_test=testp';

p_test=tramnmx(p_test,minp,maxp);

grnn_prediction_result=sim(net,p_test);

grnn_prediction_result=postmnmx(grnn_prediction_result,mint,maxt); %Prediction value of the test set

grnn_error=mean(abs(testgoal-grnn_prediction_result)); %error

save mydata grnn_prediction_result net
