# Supplementary material for: A machine learning correction for DFT non-covalent interactions based on the S22, S66 and X40 benchmark databases
Source: J Cheminform. 2016 May 3;8:24. doi: 10.1186/s13321-016-0133-7 (PMC4855356; doi:10.1186/s13321-016-0133-7)
Supplement: Supplementary file 3 — 10.1186/s13321-016-0133-7 The NCI, descriptors and errors based on M062X/6-31G*(vac). [file 13321_2016_133_MOESM3_ESM.doc]

Table S2. The NCI, descriptors and errors ^a^ based on M062X/6-31G*(vac) calculations

| NO. | Name | GRNN | NCI | D | E_lumo+1_ | N_ve_ | Error | Error new |
| --- | --- | --- | --- | --- | --- | --- | --- | --- |
| **S66** |  |  |  |  |  |  |  |  |
| 1 | Water-MeOH^b^ | -4.91 | -8.35 | 1.56 | 0.16 | 22.00 | -2.65 | 0.79 |
| 2 | Water-MeNH_2_^b^ | -6.97 | -9.82 | 3.31 | 0.16 | 22.00 | -2.79 | 0.07 |
| 3 | Water-Peptide^b^ | -7.84 | -10.66 | 4.82 | 0.11 | 38.00 | -2.44 | 0.38 |
| 4 | MeOH dimer | -6.26 | -8.81 | 3.40 | 0.16 | 28.00 | -2.95 | -0.41 |
| 5 | MeOH-MeNH_2_^b^ | -7.35 | -10.58 | 3.47 | 0.16 | 28.00 | -2.91 | 0.32 |
| 6 | MeOH-Peptide | -7.97 | -11.50 | 5.26 | 0.11 | 44.00 | -3.16 | 0.36 |
| 7 | MeOH-Water | -5.26 | -7.84 | 2.48 | 0.18 | 22.00 | -2.76 | -0.17 |
| 8 | MeNH_2_-MeOH | -2.71 | -4.46 | 3.47 | 0.16 | 28.00 | -1.34 | 0.40 |
| 9 | MeNH_2_ dimer | -4.53 | -6.25 | 1.74 | 0.16 | 28.00 | -2.03 | -0.31 |
| 10 | MeNH_2_-Peptide | -5.43 | -8.05 | 2.90 | 0.11 | 44.00 | -2.57 | 0.05 |
| 11 | MeNH_2_-Water | -6.97 | -9.82 | 3.31 | 0.16 | 22.00 | -2.42 | 0.43 |
| 12 | Peptide-MeOH | -6.62 | -8.71 | 5.68 | 0.10 | 44.00 | -2.43 | -0.33 |
| 13 | Peptide-MeNH_2_ | -7.02 | -9.85 | 6.16 | 0.10 | 44.00 | -2.28 | 0.54 |
| 14 | Peptide dimer | -8.71 | -10.68 | 7.61 | 0.10 | 60.00 | -1.96 | 0.01 |
| 15 | Peptide-Water | -6.03 | -7.86 | 5.95 | 0.10 | 38.00 | -2.66 | -0.83 |
| 16 | Uracil dimer | -17.45 | -17.70 | 7.58 | 0.01 | 84.00 | -0.25 | 0.00 |
| 17 | Water-Pyridine | -6.68 | -9.14 | 4.01 | 0.02 | 38.00 | -2.17 | 0.29 |
| 18 | MeOH-Pyridine^b^ | -6.75 | -9.86 | 4.12 | 0.02 | 44.00 | -2.35 | 0.76 |
| 19 | AcOH dimer | -18.76 | -20.28 | 0.00 | 0.06 | 48.00 | -0.86 | 0.66 |
| 20 | AcNH_2_ dimer | -16.54 | -17.83 | 0.08 | 0.09 | 50.00 | -1.30 | -0.01 |
| 21 | AcOH-Uracil | -19.74 | -20.10 | 3.56 | 0.05 | 66.00 | -0.32 | 0.04 |
| 22 | AcNH2-Uracil^b^ | -19.78 | -19.93 | 5.32 | 0.06 | 66.00 | -0.46 | -0.32 |
| 23 | Pyr dimer | -3.23 | -4.64 | 2.47 | 0.02 | 60.00 | -0.84 | 0.57 |
| 24 | Ur dimer | -9.75 | -11.80 | 4.70 | -0.01 | 84.00 | -2.05 | 0.00 |
| 25 | Ben-Pyr | -3.11 | -4.02 | 2.08 | 0.03 | 60.00 | -0.68 | 0.23 |
| 26 | Ben-Ur | -5.26 | -7.25 | 3.96 | 0.04 | 72.00 | -1.66 | 0.33 |
| 27 | Pyr-Ur | -5.43 | -8.21 | 2.25 | 0.02 | 72.00 | -1.51 | 1.27 |
| 28 | Benzene-Ethene | -1.84 | -1.96 | 0.16 | 0.04 | 42.00 | -0.59 | -0.47 |
| 29 | Ur-Ethene^b^ | -3.84 | -4.51 | 4.33 | 0.05 | 54.00 | -1.18 | -0.51 |
| 30 | Ur-Ethyne | -3.82 | -4.51 | 4.25 | 0.05 | 54.00 | -0.82 | -0.13 |
| 31 | Pyr-Ethene^b^ | -2.46 | -2.58 | 2.18 | 0.03 | 42.00 | -0.77 | -0.65 |
| 32 | Pentane dimer | -2.54 | -4.39 | 0.00 | 0.12 | 64.00 | -0.62 | 1.23 |
| 33 | Neopen-Pentane | -2.51 | -2.56 | 0.05 | 0.14 | 64.00 | 0.04 | 0.09 |
| 34 | Neopen dimer | -2.43 | -1.64 | 0.00 | 0.13 | 64.00 | 0.13 | -0.66 |
| 35 | Cyclopen-Neopen | -2.52 | -2.90 | 0.03 | 0.13 | 62.00 | -0.50 | -0.12 |
| 36 | Cyclopen-Cyclopen | -2.53 | -2.85 | 0.00 | 0.13 | 60.00 | 0.14 | 0.45 |
| 37 | Ben-Cyclopen^b^ | -3.30 | -4.43 | 0.50 | 0.03 | 60.00 | -0.92 | 0.22 |
| 38 | Ben-Neopen^b^ | -2.92 | -3.36 | 0.55 | 0.03 | 62.00 | -0.51 | -0.07 |
| 39 | Ur-Pentane^b^ | -4.60 | -6.80 | 4.13 | 0.05 | 74.00 | -1.99 | 0.22 |
| 40 | Ur-Cyclopen | -4.11 | -5.44 | 4.29 | 0.05 | 74.00 | -1.35 | -0.02 |
| 41 | Ur-Neopen | -4.00 | -4.77 | 4.27 | 0.05 | 74.00 | -1.08 | -0.31 |
| 42 | Ethene-Pentane | -2.11 | -2.81 | 0.24 | 0.12 | 44.00 | -0.82 | -0.12 |
| 43 | Ethyne-Pentane^b^ | -2.05 | -2.53 | 0.23 | 0.09 | 44.00 | -0.82 | -0.33 |
| 44 | Peptide-Pentane | -4.00 | -6.01 | 3.39 | 0.10 | 62.00 | -1.75 | 0.26 |
| 45 | Ben dimer | -2.86 | -3.11 | 0.58 | 0.03 | 60.00 | -0.28 | -0.03 |
| 46 | Pyr dimer | -3.94 | -4.09 | 4.39 | 0.01 | 60.00 | -0.59 | -0.43 |
| 47 | Ben-Pyr | -3.10 | -3.72 | 2.81 | 0.03 | 60.00 | -0.42 | 0.19 |
| 48 | Ben-Ethyne | -2.26 | -3.47 | 0.54 | 0.03 | 40.00 | -0.62 | 0.60 |
| 49 | Ethyne dimer | -1.54 | -1.99 | 0.46 | 0.09 | 20.00 | -0.45 | 0.00 |
| 50 | Ben-AcOH | -4.42 | -6.38 | 1.46 | 0.03 | 54.00 | -1.65 | 0.30 |
| 51 | Ben-AcNH_2_ | -4.09 | -6.14 | 3.63 | 0.04 | 54.00 | -1.73 | 0.31 |
| 52 | Ben-Water | -3.38 | -5.11 | 2.72 | 0.02 | 38.00 | -1.82 | -0.09 |
| 53 | Ben-MeOH^b^ | -3.26 | -5.60 | 2.10 | 0.02 | 44.00 | -1.44 | 0.91 |
| 54 | Ben-MeNH_2_^b^ | -2.85 | -4.38 | 1.86 | 0.03 | 44.00 | -1.18 | 0.35 |
| 55 | Ben-Peptide | -4.72 | -6.65 | 4.24 | 0.02 | 60.00 | -1.40 | 0.54 |
| 56 | Pyr dimer | -3.53 | -4.63 | 0.00 | 0.02 | 60.00 | -0.39 | 0.70 |
| 57 | Ethyne-Water | -3.72 | -4.68 | 2.48 | 0.12 | 18.00 | -1.76 | -0.79 |
| 58 | Ethyne-AcOH | -4.94 | -6.95 | 1.46 | 0.08 | 34.00 | -1.98 | 0.03 |
| 59 | Pentane-AcOH | -2.87 | -4.55 | 1.47 | 0.10 | 56.00 | -1.64 | 0.03 |
| 60 | Pentane-AcNH_2_ | -3.90 | -5.48 | 3.36 | 0.10 | 56.00 | -1.95 | -0.37 |
| 61 | Ben-AcOH^b^ | -3.87 | -5.23 | 1.50 | 0.03 | 54.00 | -1.48 | -0.12 |
| 62 | peptide-Ethene^b^ | -3.27 | -4.38 | 3.61 | 0.08 | 42.00 | -1.38 | -0.26 |
| 63 | Pyr-Ethyne | -3.79 | -4.59 | 3.28 | 0.02 | 40.00 | -0.49 | 0.31 |
| 64 | MeNH^2^-Pyr^b^ | -3.16 | -5.28 | 2.09 | 0.03 | 44.00 | -1.31 | 0.81 |
| **S22** |  |  |  |  |  |  |  |  |
| 65 | Adenine-Thymine | -16.34 | -17.47 | 1.44 | 0.02 | 98.00 | -1.10 | 0.03 |
| 66 | Adenine-Thymine | -12.27 | -14.60 | 3.05 | 0.01 | 98.00 | -2.37 | -0.04 |
| 67 | Ammonia dimer | -0.81 | -5.55 | 0.00 | 0.16 | 16.00 | -2.38 | 2.36 |
| 68 | Water dimer | -4.38 | -7.86 | 0.46 | 0.16 | 16.00 | -2.84 | 0.64 |
| 69 | Methane dimer^b^ | -0.53 | -0.69 | 0.00 | 0.20 | 16.00 | -0.16 | 0.00 |
| 70 | Ethene dimer | -1.51 | -2.36 | 0.00 | 0.07 | 24.00 | -0.85 | 0.00 |
| 71 | Ethene-Ethyne^b^ | -1.33 | -2.04 | 0.48 | 0.11 | 22.00 | -0.51 | 0.20 |
| 72 | Formicacid dimer | -18.44 | -19.84 | 0.00 | 0.05 | 36.00 | -1.23 | 0.17 |
| 73 | Formamide dimer | -16.11 | -17.92 | 0.01 | 0.08 | 36.00 | -1.96 | -0.15 |
| 74 | Benzene-Ammonia | -2.68 | -3.48 | 2.28 | 0.03 | 39.00 | -1.13 | -0.33 |
| 75 | Methane-Benzene^b^ | -1.85 | -1.93 | 0.23 | 0.04 | 38.00 | -0.43 | -0.35 |
| 76 | Benzene dimer | -2.79 | -2.74 | 0.58 | 0.03 | 60.00 | 0.00 | -0.05 |
| 77 | Benzene dimer | -2.95 | -3.32 | 0.00 | 0.04 | 60.00 | -0.59 | -0.22 |
| 78 | Indole-Benzene | -5.25 | -6.59 | 2.51 | 0.03 | 74.00 | -0.86 | 0.48 |
| 79 | Indole-Benzene | -5.01 | -5.30 | 1.88 | 0.05 | 74.00 | -0.08 | 0.21 |
| 80 | Pyrazine dimer | -4.35 | -5.27 | 0.22 | -0.01 | 70.00 | -0.85 | 0.07 |
| 81 | 2-pyridoxine2-aminopyridine | -16.80 | -17.50 | 2.55 | 0.02 | 72.00 | -0.79 | -0.09 |
| 82 | Phenol dimer | -6.50 | -9.41 | 3.57 | 0.04 | 72.00 | -2.36 | 0.55 |
| 83 | Uracil dimer^b^ | -9.75 | -11.82 | 4.60 | 0.00 | 84.00 | -1.70 | 0.37 |
| 84 | Uracil dimer | -20.65 | -20.40 | 0.01 | 0.00 | 84.00 | 0.25 | 0.00 |
| 85 | Benzene-HCN | -4.21 | -5.57 | 3.60 | 0.02 | 40.00 | -1.11 | 0.25 |
| **X40** |  |  |  |  |  |  |  |  |
| 86 | Methane-F_2_ | -0.58 | -0.99 | 0.02 | 0.17 | 22.00 | -0.50 | -0.09 |
| 87 | Methane-Cl_2_ | -0.67 | -1.23 | 0.25 | 0.16 | 22.00 | -0.15 | 0.41 |
| 88 | Methane-Br_2_^b^ | -1.41 | -1.62 | 0.41 | 0.05 | 22.00 | -0.32 | -0.11 |
| 89 | Methane-I_2_ | -1.36 | -1.58 | 0.49 | 0.04 | 22.00 | -0.23 | -0.02 |
| 90 | Fluoromethane-Methane^b^ | -0.65 | -1.68 | 1.69 | 0.18 | 22.00 | -0.93 | 0.10 |
| 91 | Chloromethane-Methane | -2.10 | -1.24 | 2.00 | 0.13 | 22.00 | -0.26 | -1.12 |
| 92 | Trifluoromethane-Methane^b^ | -1.97 | -2.08 | 1.65 | 0.17 | 34.00 | -1.39 | -1.27 |
| 93 | Trichloromethane-Methane | -1.42 | -1.52 | 1.31 | 0.06 | 34.00 | -0.37 | -0.27 |
| 94 | Fluoromethane-Fluoromethane | -1.92 | -2.72 | 3.90 | 0.17 | 28.00 | -1.07 | -0.28 |
| 95 | Chloromethane-Chloromethane | -1.69 | -1.31 | 4.39 | 0.11 | 28.00 | 0.03 | -0.35 |
| 96 | BenF_3_-Ben | -4.40 | -5.41 | 0.32 | 0.03 | 78.00 | -1.01 | 0.00 |
| 97 | BenF_6_-Ben | -6.10 | -7.41 | 0.50 | 0.02 | 96.00 | -1.29 | 0.03 |
| 98 | Chloromethane-Formaldehyde | -1.18 | -1.89 | 3.12 | 0.09 | 26.00 | -0.72 | -0.01 |
| 99 | Bromomethane-Formaldehyde^b^ | -2.25 | -2.64 | 2.85 | 0.04 | 26.00 | -0.91 | -0.53 |
| 100 | Iodomethane-Formaldehyde | -2.50 | -3.48 | 2.56 | 0.02 | 26.00 | -1.10 | -0.12 |
| 101 | F_3_chloromethane-Formaldehyde | -2.31 | -3.09 | 2.48 | 0.07 | 44.00 | -0.84 | -0.07 |
| 102 | F_3_bromomethane-Formaldehyde | -3.23 | -4.12 | 2.76 | 0.02 | 44.00 | -1.01 | -0.13 |
| 103 | F_3_iodomethane-Formaldehyde^b^ | -4.12 | -5.32 | 3.47 | -0.01 | 44.00 | -1.24 | -0.04 |
| 104 | BenCl-Acetone | -3.06 | -3.98 | 2.36 | 0.03 | 60.00 | -2.49 | -1.57 |
| 105 | BenBr-Acetone^b^ | -3.20 | -4.73 | 2.60 | 0.03 | 60.00 | -2.30 | -0.78 |
| 106 | BenI-Acetone | -3.55 | -6.00 | 2.85 | 0.03 | 60.00 | -2.54 | -0.09 |
| 107 | BenCl-NMe_3_ | -2.81 | -2.88 | 0.71 | 0.03 | 62.00 | -0.76 | -0.70 |
| 108 | BenBr- NMe_3_^b^ | -3.17 | -3.99 | 0.12 | 0.03 | 62.00 | -0.22 | 0.60 |
| 109 | BenI- NMe_3_ | -4.86 | -6.57 | 1.11 | 0.04 | 62.00 | -0.77 | 0.94 |
| 110 | BenBr-MeSH | -2.46 | -0.70 | 2.80 | 0.02 | 50.00 | 1.62 | -0.14 |
| 111 | BenI-MeSH^b^ | -2.53 | -1.71 | 2.22 | 0.02 | 50.00 | 1.37 | 0.54 |
| 112 | CH_3_Br-Ben | -1.96 | -0.88 | 1.53 | 0.04 | 44.00 | 0.93 | -0.14 |
| 113 | CH_3_I-Ben | -2.09 | 0.25 | 1.04 | 0.04 | 44.00 | 2.73 | 0.40 |
| 114 | CF3Br-Ben^b^ | -2.82 | -2.76 | 1.09 | 0.03 | 62.00 | 0.35 | 0.29 |
| 115 | CF_3_I-Ben | -3.09 | -3.52 | 1.83 | 0.03 | 62.00 | 0.40 | 0.82 |
| 116 | TrifluorometOH-Water | -9.72 | -14.64 | 2.61 | 0.17 | 40.00 | -4.97 | -0.05 |
| 117 | TrichlorometOH-Water | -10.41 | -16.05 | 2.99 | 0.06 | 40.00 | -5.64 | 0.00 |
| 118 | HF-MeOH | -9.16 | -12.56 | 3.43 | 0.17 | 22.00 | -2.97 | 0.43 |
| 119 | HF-MeNH_2_ | -14.26 | -16.44 | 4.34 | 0.18 | 32.00 | -2.13 | 0.06 |
| 120 | Methanol-Fluoromethane | -3.93 | -6.52 | 0.54 | 0.17 | 28.00 | -2.63 | -0.04 |
| 121 | Methanol-Chloromethane | -3.81 | -4.87 | 0.94 | 0.13 | 28.00 | -1.10 | -0.03 |

- ^a^ The errors regards to CCSD(T)/CBS benchmark NCI valules.
- ^b^ The molecules in the test set.
