# Supplementary material for: A machine learning correction for DFT non-covalent interactions based on the S22, S66 and X40 benchmark databases
Source: J Cheminform. 2016 May 3;8:24. doi: 10.1186/s13321-016-0133-7 (PMC4855356; doi:10.1186/s13321-016-0133-7)
Supplement: Supplementary file 5 — 10.1186/s13321-016-0133-7 The NCI, descriptors and errors based on M062X/6-31G*(ε=4.20) calculations. [file 13321_2016_133_MOESM5_ESM.docx]

Table S4. The NCI, descriptors and errors ^a^ based on M062X/6-31G*(ε=4.20)calculations

| NO. | Name | GRNN | NCI | D | E_lumo+1_ | N_ve_ | Error | Error new |
| --- | --- | --- | --- | --- | --- | --- | --- | --- |
| **S66** |  |  |  |  |  |  |  |  |
| 1 | Water-MeOH^b^ | -5.69 | -7.03 | 2.98 | 0.16 | 22.00 | -1.33 | 0.01 |
| 2 | Water-MeNH_2_^b^ | -7.09 | -8.50 | 4.79 | 0.16 | 22.00 | -1.47 | -0.05 |
| 3 | Water-Peptide^b^ | -7.28 | -8.78 | 6.68 | 0.12 | 38.00 | -0.56 | 0.94 |
| 4 | MeOH dimer | -5.88 | -7.68 | 3.51 | 0.16 | 28.00 | -1.83 | -0.02 |
| 5 | MeOH-MeNH_2_^b^ | -7.80 | -9.87 | 4.56 | 0.16 | 28.00 | -2.20 | -0.13 |
| 6 | MeOH-Peptide | -7.79 | -9.79 | 6.71 | 0.12 | 44.00 | -1.45 | 0.54 |
| 7 | MeOH-Water | -5.68 | -7.30 | 3.91 | 0.18 | 22.00 | -2.21 | -0.60 |
| 8 | MeNH_2_-MeOH | -3.24 | -4.44 | 0.89 | 0.15 | 28.00 | -1.33 | -0.12 |
| 9 | MeNH_2_ dimer | -3.73 | -4.79 | 2.86 | 0.16 | 28.00 | -0.56 | 0.49 |
| 10 | MeNH_2_-Peptide | -5.22 | -6.13 | 4.04 | 0.12 | 44.00 | -0.65 | 0.26 |
| 11 | MeNH_2_-Water | -7.05 | -8.80 | 4.53 | 0.17 | 22.00 | -1.40 | 0.35 |
| 12 | Peptide-MeOH | -6.50 | -7.45 | 6.63 | 0.10 | 44.00 | -1.17 | -0.22 |
| 13 | Peptide-MeNH_2_ | -7.14 | -8.90 | 7.48 | 0.11 | 44.00 | -1.33 | 0.42 |
| 14 | Peptide dimer | -8.72 | -9.29 | 9.40 | 0.09 | 60.00 | -0.56 | 0.00 |
| 15 | Peptide-Water | -5.73 | -7.01 | 7.69 | 0.10 | 38.00 | -1.81 | -0.53 |
| 16 | Uracil dimer | -17.45 | -13.84 | 9.25 | 0.00 | 84.00 | 3.60 | 0.00 |
| 17 | Water-Pyridine | -6.86 | -7.52 | 5.22 | 0.02 | 38.00 | -0.55 | 0.11 |
| 18 | MeOH-Pyridine^b^ | -6.89 | -8.48 | 0.02 | 0.02 | 44.00 | -0.97 | 0.62 |
| 19 | AcOH dimer | -19.22 | -16.82 | 0.01 | 0.05 | 48.00 | 2.59 | 0.20 |
| 20 | AcNH_2_ dimer | -16.52 | -13.57 | 0.23 | 0.08 | 50.00 | 2.96 | 0.01 |
| 21 | AcOH-Uracil | -19.62 | -15.63 | 4.30 | 0.05 | 66.00 | 4.15 | 0.16 |
| 22 | AcNH2-Uracil^b^ | -19.74 | -14.82 | 6.43 | 0.05 | 66.00 | 4.65 | -0.28 |
| 23 | Pyr dimer | -3.40 | -3.96 | 3.16 | 0.02 | 60.00 | -0.16 | 0.40 |
| 24 | Ur dimer | -9.39 | -8.07 | 4.25 | 0.00 | 84.00 | 1.68 | 0.36 |
| 25 | Ben-Pyr | -3.21 | -3.29 | 2.65 | 0.03 | 60.00 | 0.06 | 0.13 |
| 26 | Ben-Ur | -4.97 | -6.32 | 5.11 | 0.03 | 72.00 | -0.73 | 0.62 |
| 27 | Pyr-Ur | -6.28 | -6.66 | 3.70 | 0.01 | 72.00 | 0.04 | 0.43 |
| 28 | Benzene-Ethene | -1.86 | -1.47 | 0.13 | 0.04 | 42.00 | -0.10 | -0.49 |
| 29 | Ur-Ethene^b^ | -4.27 | -3.96 | 5.36 | 0.05 | 54.00 | -0.63 | -0.94 |
| 30 | Ur-Ethyne | -4.20 | -3.45 | 5.30 | 0.05 | 54.00 | 0.25 | -0.51 |
| 31 | Pyr-Ethene^b^ | -2.57 | -2.28 | 2.68 | 0.03 | 42.00 | -0.47 | -0.76 |
| 32 | Pentane dimer | -2.55 | -4.36 | 0.00 | 0.12 | 64.00 | -0.60 | 1.22 |
| 33 | Neopen-Pentane | -2.44 | -2.54 | 0.07 | 0.14 | 64.00 | 0.06 | 0.17 |
| 34 | Neopen dimer | -2.29 | -1.54 | 0.00 | 0.13 | 64.00 | 0.23 | -0.53 |
| 35 | Cyclopen-Neopen | -2.47 | -2.85 | 0.03 | 0.13 | 62.00 | -0.45 | -0.07 |
| 36 | Cyclopen-Cyclopen | -2.48 | -2.81 | 0.00 | 0.13 | 60.00 | 0.17 | 0.50 |
| 37 | Ben-Cyclopen^b^ | -3.16 | -4.17 | 0.45 | 0.03 | 60.00 | -0.66 | 0.36 |
| 38 | Ben-Neopen^b^ | -2.94 | -3.18 | 0.46 | 0.03 | 62.00 | -0.33 | -0.09 |
| 39 | Ur-Pentane^b^ | -4.56 | -6.36 | 5.27 | 0.05 | 74.00 | -1.55 | 0.25 |
| 40 | Ur-Cyclopen | -4.20 | -5.22 | 5.37 | 0.05 | 74.00 | -1.13 | -0.11 |
| 41 | Ur-Neopen | -4.02 | -4.38 | 5.35 | 0.05 | 74.00 | -0.69 | -0.33 |
| 42 | Ethene-Pentane | -2.11 | -2.65 | 0.22 | 0.12 | 44.00 | -0.66 | -0.12 |
| 43 | Ethyne-Pentane^b^ | -2.03 | -2.24 | 0.25 | 0.09 | 44.00 | -0.52 | -0.32 |
| 44 | Peptide-Pentane | -3.98 | -5.47 | 4.36 | 0.10 | 62.00 | -1.22 | 0.28 |
| 45 | Ben dimer | -2.91 | -2.91 | 0.47 | 0.03 | 60.00 | -0.08 | -0.08 |
| 46 | Pyr dimer | -3.78 | -3.46 | 5.14 | 0.02 | 60.00 | 0.05 | -0.28 |
| 47 | Ben-Pyr | -3.17 | -3.23 | 3.17 | 0.02 | 60.00 | 0.06 | 0.12 |
| 48 | Ben-Ethyne | -2.31 | -2.99 | 0.49 | 0.03 | 40.00 | -0.13 | 0.55 |
| 49 | Ethyne dimer | -1.58 | -1.52 | 0.39 | 0.09 | 20.00 | 0.02 | -0.04 |
| 50 | Ben-AcOH | -4.13 | -5.13 | 1.91 | 0.03 | 54.00 | -0.40 | 0.60 |
| 51 | Ben-AcNH_2_ | -4.13 | -4.41 | 4.46 | 0.04 | 54.00 | 0.00 | 0.28 |
| 52 | Ben-Water | -3.32 | -4.23 | 2.88 | 0.03 | 38.00 | -0.94 | -0.03 |
| 53 | Ben-MeOH^b^ | -3.45 | -4.96 | 2.34 | 0.03 | 44.00 | -0.80 | 0.72 |
| 54 | Ben-MeNH_2_^b^ | -2.89 | -3.93 | 2.01 | 0.03 | 44.00 | -0.73 | 0.31 |
| 55 | Ben-Peptide | -4.79 | -5.90 | 5.05 | 0.02 | 60.00 | -0.65 | 0.46 |
| 56 | Pyr dimer | -3.24 | -3.21 | 0.00 | 0.02 | 60.00 | 1.03 | 1.00 |
| 57 | Ethyne-Water | -1.74 | -4.04 | 2.69 | 0.11 | 18.00 | -1.11 | 1.18 |
| 58 | Ethyne-AcOH | -4.80 | -4.96 | 1.76 | 0.08 | 34.00 | 0.00 | 0.16 |
| 59 | Pentane-AcOH | -2.90 | -4.17 | 1.88 | 0.10 | 56.00 | -1.27 | 0.01 |
| 60 | Pentane-AcNH_2_ | -3.89 | -4.89 | 4.35 | 0.10 | 56.00 | -1.36 | -0.36 |
| 61 | Ben-AcOH^b^ | -3.79 | -4.50 | 1.89 | 0.03 | 54.00 | -0.75 | -0.04 |
| 62 | peptide-Ethene^b^ | -3.26 | -3.58 | 4.54 | 0.08 | 42.00 | -0.58 | -0.26 |
| 63 | Pyr-Ethyne | -3.75 | -3.82 | 3.66 | 0.02 | 40.00 | 0.29 | 0.36 |
| 64 | MeNH^2^-Pyr^b^ | -3.55 | -4.34 | 3.14 | 0.02 | 44.00 | -0.38 | 0.41 |
| **S22** |  |  |  |  |  |  |  |  |
| 65 | Adenine-Thymine | -16.39 | -14.11 | 1.94 | 0.02 | 98.00 | 2.26 | -0.02 |
| 66 | Adenine-Thymine | -11.86 | -11.41 | 4.03 | 0.01 | 98.00 | 0.82 | 0.37 |
| 67 | Ammonia dimer | -2.34 | -3.25 | 0.17 | 0.16 | 16.00 | -0.08 | 0.83 |
| 68 | Water dimer | -5.52 | -6.68 | 3.42 | 0.17 | 16.00 | -1.66 | -0.50 |
| 69 | Methane dimer^b^ | -0.57 | -0.67 | 0.00 | 0.20 | 16.00 | -0.14 | -0.04 |
| 70 | Ethene dimer | -1.42 | -2.01 | 0.00 | 0.06 | 24.00 | -0.50 | 0.09 |
| 71 | Ethene-Ethyne^b^ | -1.72 | -1.63 | 0.41 | 0.10 | 22.00 | -0.10 | -0.19 |
| 72 | Formicacid dimer | -18.66 | -16.30 | 0.00 | 0.05 | 36.00 | 2.31 | -0.05 |
| 73 | Formamide dimer | -16.10 | -13.51 | 0.01 | 0.08 | 36.00 | 2.45 | -0.14 |
| 74 | Benzene-Ammonia | -2.55 | -2.94 | 2.38 | 0.03 | 39.00 | -0.59 | -0.20 |
| 75 | Methane-Benzene^b^ | -1.90 | -1.71 | 0.19 | 0.04 | 38.00 | -0.21 | -0.40 |
| 76 | Benzene dimer | -2.87 | -2.53 | 0.44 | 0.03 | 60.00 | 0.21 | -0.13 |
| 77 | Benzene dimer | -2.88 | -2.53 | 0.00 | 0.04 | 60.00 | 0.20 | -0.15 |
| 78 | Indole-Benzene | -5.15 | -5.20 | 3.44 | 0.02 | 74.00 | 0.53 | 0.58 |
| 79 | Indole-Benzene | -4.67 | -3.90 | 2.58 | 0.04 | 74.00 | 1.32 | 0.55 |
| 80 | Pyrazine dimer | -4.33 | -4.57 | 0.15 | -0.01 | 70.00 | -0.15 | 0.09 |
| 81 | 2-pyridoxine2-aminopyridine | -16.85 | -13.76 | 3.63 | 0.02 | 72.00 | 2.95 | -0.14 |
| 82 | Phenol dimer | -6.04 | -8.34 | 4.16 | 0.04 | 72.00 | -1.29 | 1.01 |
| 83 | Uracil dimer^b^ | -9.43 | -8.11 | 4.40 | 0.00 | 84.00 | 2.01 | 0.69 |
| 84 | Uracil dimer | -20.63 | -14.72 | 0.09 | 0.00 | 84.00 | 5.93 | 0.02 |
| 85 | Benzene-HCN | -3.97 | -4.37 | 3.96 | 0.02 | 40.00 | 0.09 | 0.49 |
| **X40** |  |  |  |  |  |  |  |  |
| 86 | Methane-F_2_ | -0.86 | -0.95 | 0.02 | 0.17 | 22.00 | -0.46 | -0.37 |
| 87 | Methane-Cl_2_ | -0.97 | -1.10 | 0.23 | 0.17 | 22.00 | -0.02 | 0.11 |
| 88 | Methane-Br_2_^b^ | -1.37 | -1.39 | 0.37 | 0.06 | 22.00 | -0.09 | -0.07 |
| 89 | Methane-I_2_ | -1.36 | -1.30 | 0.41 | 0.04 | 22.00 | 0.04 | -0.01 |
| 90 | Fluoromethane-Methane^b^ | -0.85 | -1.24 | 1.87 | 0.19 | 22.00 | -0.49 | -0.10 |
| 91 | Chloromethane-Methane | -1.10 | -1.13 | 2.39 | 0.13 | 22.00 | -0.15 | -0.12 |
| 92 | Trifluoromethane-Methane^b^ | -1.15 | -2.08 | 1.89 | 0.18 | 34.00 | -1.39 | -0.46 |
| 93 | Trichloromethane-Methane | -1.47 | -1.39 | 1.55 | 0.06 | 34.00 | -0.24 | -0.33 |
| 94 | Fluoromethane-Fluoromethane | -1.64 | -2.17 | 4.24 | 0.16 | 28.00 | -0.52 | 0.01 |
| 95 | Chloromethane-Chloromethane | -1.32 | -0.81 | 4.94 | 0.10 | 28.00 | 0.53 | 0.01 |
| 96 | BenF_3_-Ben | -4.35 | -4.77 | 0.24 | 0.03 | 78.00 | -0.37 | 0.06 |
| 97 | BenF_6_-Ben | -6.10 | -6.60 | 0.36 | 0.02 | 96.00 | -0.48 | 0.03 |
| 98 | Chloromethane-Formaldehyde | -1.19 | -1.76 | 3.61 | 0.08 | 26.00 | -0.59 | -0.02 |
| 99 | Bromomethane-Formaldehyde^b^ | -2.48 | -2.32 | 3.25 | 0.04 | 26.00 | -0.60 | -0.76 |
| 100 | Iodomethane-Formaldehyde | -2.48 | -2.97 | 2.81 | 0.01 | 26.00 | -0.59 | -0.10 |
| 101 | F_3_chloromethane-Formaldehyde | -2.41 | -2.74 | 2.88 | 0.07 | 44.00 | -0.49 | -0.17 |
| 102 | F_3_bromomethane-Formaldehyde | -3.45 | -3.67 | 3.23 | 0.02 | 44.00 | -0.57 | -0.34 |
| 103 | F_3_iodomethane-Formaldehyde^b^ | -4.04 | -4.83 | 4.17 | 0.00 | 44.00 | -0.75 | 0.04 |
| 104 | BenCl-Acetone | -2.70 | -1.95 | 2.84 | 0.03 | 60.00 | -0.46 | -1.21 |
| 105 | BenBr-Acetone^b^ | -3.53 | -4.42 | 2.86 | 0.03 | 60.00 | -2.00 | -1.11 |
| 106 | BenI-Acetone | -3.78 | -5.46 | 3.19 | 0.02 | 60.00 | -2.00 | -0.32 |
| 107 | BenCl-NMe_3_ | -2.89 | -2.52 | 1.07 | 0.03 | 62.00 | -0.40 | -0.77 |
| 108 | BenBr- NMe_3_^b^ | -3.10 | -3.50 | 0.07 | 0.03 | 62.00 | 0.27 | 0.68 |
| 109 | BenI- NMe_3_ | -4.99 | -6.12 | 1.32 | 0.03 | 62.00 | -0.31 | 0.82 |
| 110 | BenBr-MeSH | -2.29 | -0.28 | 3.35 | 0.02 | 50.00 | 2.04 | 0.03 |
| 111 | BenI-MeSH^b^ | -2.31 | -1.14 | 2.63 | 0.02 | 50.00 | 1.94 | 0.76 |
| 112 | CH_3_Br-Ben | -2.08 | -0.87 | 1.89 | 0.04 | 44.00 | 0.94 | -0.26 |
| 113 | CH_3_I-Ben | -2.13 | -1.35 | 1.41 | 0.03 | 44.00 | 1.13 | 0.36 |
| 114 | CF3Br-Ben^b^ | -2.85 | -2.41 | 1.04 | 0.03 | 62.00 | 0.70 | 0.26 |
| 115 | CF_3_I-Ben | -3.14 | -2.94 | 1.93 | 0.03 | 62.00 | 0.97 | 0.78 |
| 116 | TrifluorometOH-Water | -9.68 | -12.33 | 4.70 | 0.19 | 40.00 | -2.66 | -0.01 |
| 117 | TrichlorometOH-Water | -10.41 | -13.95 | 4.56 | 0.06 | 40.00 | -3.54 | 0.00 |
| 118 | HF-MeOH | -8.92 | -10.58 | 5.12 | 0.17 | 22.00 | -0.98 | 0.67 |
| 119 | HF-MeNH_2_ | -14.30 | -16.56 | 5.31 | 0.18 | 32.00 | -2.24 | 0.02 |
| 120 | Methanol-Fluoromethane | -3.09 | -5.01 | 1.36 | 0.17 | 28.00 | -1.12 | 0.81 |
| 121 | Methanol-Chloromethane | -3.37 | -2.95 | 0.98 | 0.13 | 28.00 | 0.83 | 0.41 |

^a^ The errors regards to CCSD(T)/CBS benchmark NCI valules.

^b^The molecules in the test set.
