# Supplementary material for: A machine learning correction for DFT non-covalent interactions based on the S22, S66 and X40 benchmark databases
Source: J Cheminform. 2016 May 3;8:24. doi: 10.1186/s13321-016-0133-7 (PMC4855356; doi:10.1186/s13321-016-0133-7)
Supplement: Supplementary file 6 — 10.1186/s13321-016-0133-7 The NCI, descriptors and errors based on M062X/6-31+G* calculations. [file 13321_2016_133_MOESM6_ESM.docx]

Table S5. The NCI, descriptors and errors ^a^ based on M062X/6-31+G* calculations

| NO. | Name | GRNN | NCI | Arrangement | ESE | N_ve_ | Error | Error new |
| --- | --- | --- | --- | --- | --- | --- | --- | --- |
| **S66** |  |  |  |  |  |  |  |  |
| 1 | Water-MeOH^b^ | -5.19 | -5.54 | 1.00 | 325.42 | 22.00 | 0.16 | 0.51 |
| 2 | Water-MeNH_2_^b^ | -7.37 | -7.95 | 1.00 | 334.93 | 22.00 | -0.91 | -0.34 |
| 3 | Water-Peptide | -7.38 | -6.86 | 1.00 | 914.55 | 38.00 | 1.36 | 0.84 |
| 4 | MeOH dimer | -5.75 | -5.85 | 1.00 | 514.85 | 28.00 | 0.00 | 0.10 |
| 5 | MeOH-MeNH_2_^b^ | -7.38 | -8.58 | 1.00 | 505.81 | 28.00 | -0.91 | 0.28 |
| 6 | MeOH-Peptide^b^ | -7.39 | -6.80 | 1.00 | 1286.13 | 44.00 | 1.54 | 0.94 |
| 7 | MeOH-Water | -5.18 | -5.53 | 1.00 | 318.61 | 22.00 | -0.44 | -0.10 |
| 8 | MeNH_2_-MeOH^b^ | -3.00 | -2.67 | 1.00 | 536.27 | 28.00 | 0.44 | 0.11 |
| 9 | MeNH_2_ dimer | -3.50 | -3.34 | 1.00 | 554.04 | 28.00 | 0.88 | 0.73 |
| 10 | MeNH_2_-Peptide | -4.67 | -3.48 | 1.00 | 1070.08 | 44.00 | 2.00 | 0.81 |
| 11 | MeNH_2_-Water | -7.37 | -7.95 | 1.00 | 331.36 | 22.00 | -0.55 | 0.03 |
| 12 | Peptide-MeOH | -6.30 | -5.26 | 1.00 | 1154.76 | 44.00 | 1.02 | -0.02 |
| 13 | Peptide-MeNH_2_ | -7.46 | -7.00 | 1.00 | 1181.47 | 44.00 | 0.56 | 0.10 |
| 14 | Peptide dimer | -8.64 | -6.31 | 1.00 | 2409.91 | 60.00 | 2.41 | 0.08 |
| 15 | Peptide-Water^b^ | -6.16 | -5.25 | 1.00 | 852.32 | 38.00 | -0.05 | -0.96 |
| 16 | Uracil dimer | -17.45 | -9.90 | 0.00 | 5116.33 | 84.00 | 7.54 | 0.00 |
| 17 | Water-Pyridine | -7.33 | -6.28 | 0.00 | 966.28 | 38.00 | 0.69 | -0.35 |
| 18 | MeOH-Pyridine | -7.51 | -6.78 | 2.00 | 1376.68 | 44.00 | 0.73 | 0.00 |
| 19 | AcOH dimer | -19.41 | -12.72 | 0.00 | 1467.23 | 48.00 | 6.70 | 0.00 |
| 20 | AcNH_2_ dimer | -16.52 | -8.46 | 0.00 | 1627.00 | 50.00 | 8.07 | 0.00 |
| 21 | AcOH-Uracil | -19.78 | -11.44 | 0.00 | 3018.08 | 66.00 | 8.35 | 0.00 |
| 22 | AcNH2-Uracil^b^ | -19.28 | -9.77 | 0.00 | 3072.86 | 66.00 | 9.70 | 0.18 |
| 23 | Pyr dimer | -3.84 | -3.28 | 1.00 | 1843.28 | 60.00 | 0.52 | -0.03 |
| 24 | Ur dimer | -9.70 | -6.17 | 1.00 | 2687.87 | 84.00 | 3.58 | 0.05 |
| 25 | Ben-Pyr^b^ | -3.48 | -2.79 | 1.00 | 1936.49 | 60.00 | 0.55 | -0.14 |
| 26 | Ben-Ur | -5.88 | -5.43 | 1.00 | 2258.08 | 72.00 | 0.16 | -0.29 |
| 27 | Pyr-Ur | -5.95 | -5.52 | 1.00 | 2214.47 | 72.00 | 1.18 | 0.75 |
| 28 | Benzene-Ethene | -2.04 | -1.11 | 1.00 | 1055.57 | 42.00 | 0.25 | -0.68 |
| 29 | Ur-Ethene | -3.65 | -3.21 | 1.00 | 1370.35 | 54.00 | 0.12 | -0.32 |
| 30 | Ur-Ethyne | -3.58 | -2.64 | 1.00 | 1306.76 | 54.00 | 1.05 | 0.11 |
| 31 | Pyr-Ethene^b^ | -2.41 | -1.79 | 1.00 | 980.55 | 42.00 | 0.02 | -0.60 |
| 32 | Pentane dimer | -3.88 | -4.03 | 1.00 | 2480.41 | 64.00 | -0.26 | -0.12 |
| 33 | Neopen-Pentane | -2.67 | -2.15 | 1.00 | 2725.68 | 64.00 | 0.45 | -0.06 |
| 34 | Neopen dimer | -2.66 | -1.15 | 1.00 | 3053.91 | 64.00 | 0.62 | -0.90 |
| 35 | Cyclopen-Neopen | -2.43 | -2.46 | 1.00 | 5817.95 | 62.00 | -0.07 | -0.03 |
| 36 | Cyclopen-Cyclopen^b^ | -3.21 | -2.37 | 1.00 | 2076.24 | 60.00 | 0.62 | -0.22 |
| 37 | Ben-Cyclopen^b^ | -3.90 | -3.36 | 1.00 | 1991.88 | 60.00 | 0.15 | -0.39 |
| 38 | Ben-Neopen^b^ | -3.00 | -2.56 | 1.00 | 2456.69 | 62.00 | 0.29 | -0.15 |
| 39 | Ur-Pentane | -4.82 | -4.65 | 1.00 | 2580.47 | 74.00 | 0.16 | -0.01 |
| 40 | Ur-Cyclopen | -4.44 | -3.86 | 1.00 | 2379.58 | 74.00 | 0.24 | -0.35 |
| 41 | Ur-Neopen | -3.91 | -3.20 | 1.00 | 2903.19 | 74.00 | 0.49 | -0.23 |
| 42 | Ethene-Pentane | -2.38 | -1.91 | 1.00 | 1326.82 | 44.00 | 0.08 | -0.39 |
| 43 | Ethyne-Pentane^b^ | -2.16 | -1.41 | 1.00 | 1251.53 | 44.00 | 0.31 | -0.45 |
| 44 | Peptide-Pentane | -4.50 | -4.12 | 1.00 | 2088.22 | 62.00 | 0.13 | -0.24 |
| 45 | Ben dimer^b^ | -2.89 | -2.46 | 1.00 | 2679.05 | 60.00 | 0.37 | -0.06 |
| 46 | Pyr dimer^b^ | -3.04 | -2.43 | 2.00 | 2585.00 | 60.00 | 1.08 | 0.47 |
| 47 | Ben-Pyr | -3.05 | -2.61 | 2.00 | 2627.94 | 60.00 | 0.68 | 0.25 |
| 48 | Ben-Ethyne | -4.17 | -2.32 | 2.00 | 1466.79 | 40.00 | 0.54 | -1.31 |
| 49 | Ethyne dimer | -1.54 | -0.76 | 2.00 | 589.57 | 20.00 | 0.78 | 0.00 |
| 50 | Ben-AcOH | -3.94 | -3.60 | 1.00 | 1774.14 | 54.00 | 1.13 | 0.79 |
| 51 | Ben-AcNH_2_ | -4.09 | -1.81 | 1.00 | 2275.45 | 54.00 | 2.59 | 0.31 |
| 52 | Ben-Water | -2.94 | -2.72 | 1.00 | 753.57 | 38.00 | 0.57 | 0.34 |
| 53 | Ben-MeOH | -4.46 | -3.83 | 1.00 | 1483.32 | 44.00 | 0.34 | -0.29 |
| 54 | Ben-MeNH_2_^b^ | -3.81 | -3.10 | 1.00 | 1686.32 | 44.00 | 0.10 | -0.61 |
| 55 | Ben-Peptide | -4.69 | -4.36 | 1.00 | 1917.62 | 60.00 | 0.90 | 0.57 |
| 56 | Pyr dimer | -4.10 | -1.49 | 0.00 | 3477.03 | 60.00 | 2.75 | 0.13 |
| 57 | Ethyne-Water | -2.93 | -2.63 | 0.00 | 381.40 | 18.00 | 0.29 | 0.00 |
| 58 | Ethyne-AcOH | -4.96 | -2.70 | 0.00 | 850.75 | 34.00 | 2.27 | 0.01 |
| 59 | Pentane-AcOH | -3.61 | -2.84 | 1.00 | 1815.59 | 56.00 | 0.06 | -0.70 |
| 60 | Pentane-AcNH_2_^b^ | -3.77 | -3.24 | 1.00 | 1749.73 | 56.00 | 0.29 | -0.24 |
| 61 | Ben-AcOH | -3.78 | -3.43 | 1.00 | 1571.10 | 54.00 | 0.32 | -0.03 |
| 62 | peptide-Ethene | -2.51 | -1.98 | 1.00 | 1038.50 | 42.00 | 1.02 | 0.49 |
| 63 | Pyr-Ethyne | -4.11 | -2.55 | 0.00 | 1532.22 | 40.00 | 1.55 | -0.01 |
| 64 | MeNH^2^-Pyr^b^ | -4.40 | -3.30 | 1.00 | 1090.19 | 44.00 | 0.66 | -0.43 |
| **S22** |  |  |  |  |  |  |  |  |
| 65 | Adenine-Thymine | -16.37 | -9.85 | 0.00 | 6723.70 | 98.00 | 6.52 | 0.00 |
| 66 | Adenine-Thymine | -12.23 | -9.17 | 1.00 | 3517.21 | 98.00 | 3.06 | 0.00 |
| 67 | Ammonia dimer | -1.83 | -1.08 | 1.00 | 241.15 | 16.00 | 2.09 | 1.34 |
| 68 | Water dimer | -5.03 | -5.30 | 1.00 | 182.66 | 16.00 | -0.28 | -0.01 |
| 69 | Methane dimer | -1.52 | -0.54 | 1.00 | 294.09 | 16.00 | -0.01 | -0.99 |
| 70 | Ethene dimer | -1.54 | -1.02 | 2.00 | 540.73 | 24.00 | 0.49 | -0.03 |
| 71 | Ethene-Ethyne^b^ | -1.54 | -0.85 | 2.00 | 644.91 | 22.00 | 0.68 | -0.01 |
| 72 | Formicacid dimer | -18.61 | -12.10 | 0.00 | 667.32 | 36.00 | 6.51 | 0.00 |
| 73 | Formamide dimer | -15.61 | -8.13 | 0.00 | 762.69 | 36.00 | 7.83 | 0.35 |
| 74 | Benzene-Ammonia | -2.68 | -2.15 | 1.00 | 805.15 | 39.00 | 0.20 | -0.33 |
| 75 | Methane-Benzene^b^ | -2.12 | -1.35 | 1.00 | 875.76 | 38.00 | 0.15 | -0.62 |
| 76 | Benzene dimer | -3.02 | -2.22 | 2.00 | 2704.13 | 60.00 | 0.52 | -0.28 |
| 77 | Benzene dimer | -3.09 | -2.00 | 1.00 | 2022.36 | 60.00 | 0.73 | -0.36 |
| 78 | Indole-Benzene | -5.73 | -3.69 | 2.00 | 3591.36 | 74.00 | 2.04 | 0.00 |
| 79 | Indole-Benzene | -4.16 | -3.51 | 1.00 | 2657.79 | 74.00 | 1.71 | 1.06 |
| 80 | Pyrazine dimer | -4.45 | -3.72 | 1.00 | 1765.73 | 70.00 | 0.70 | -0.03 |
| 81 | 2-pyridoxine2-aminopyridine | -16.71 | -9.60 | 0.00 | 3681.21 | 72.00 | 7.11 | 0.00 |
| 82 | Phenol dimer | -7.05 | -6.62 | 1.00 | 3623.48 | 72.00 | 0.43 | 0.00 |
| 83 | Uracil dimer^b^ | -9.70 | -6.18 | 1.00 | 2680.28 | 84.00 | 3.94 | 0.42 |
| 84 | Uracil dimer | -17.45 | -10.06 | 0.00 | 5566.93 | 84.00 | 10.59 | 3.20 |
| 85 | Benzene-HCN | -4.46 | -3.03 | 2.00 | 1014.79 | 40.00 | 1.43 | 0.00 |
| **X40** |  |  |  |  |  |  |  |  |
| 86 | Methane-F_2_ | -0.98 | -0.45 | 1.00 | 440.86 | 22.00 | 0.04 | -0.49 |
| 87 | Methane-Cl_2_ | -1.08 | -0.95 | 1.00 | 2691.27 | 22.00 | 0.13 | 0.00 |
| 88 | Methane-Br_2_ | -1.38 | -1.32 | 1.00 | 732.34 | 22.00 | -0.02 | -0.08 |
| 89 | Methane-I_2_ | -1.41 | -1.06 | 1.00 | 1498.65 | 22.00 | 0.28 | -0.07 |
| 90 | Fluoromethane-Methane^b^ | -1.03 | -0.70 | 1.00 | 374.79 | 22.00 | 0.05 | -0.28 |
| 91 | Chloromethane-Methane | -1.14 | -0.97 | 1.00 | 496.35 | 22.00 | 0.01 | -0.16 |
| 92 | Trifluoromethane-Methane | -1.09 | -0.59 | 1.00 | 568.78 | 34.00 | 0.10 | -0.40 |
| 93 | Trichloromethane-Methane | -1.32 | -1.20 | 1.00 | 1333.55 | 34.00 | -0.05 | -0.17 |
| 94 | Fluoromethane-Fluoromethane | -2.07 | -0.81 | 1.00 | 767.56 | 28.00 | 0.84 | -0.42 |
| 95 | Chloromethane-Chloromethane | -1.33 | -0.57 | 1.00 | 1693.41 | 28.00 | 0.76 | 0.01 |
| 96 | BenF_3_-Ben | -4.37 | -4.10 | 1.00 | 2818.07 | 78.00 | 0.31 | 0.04 |
| 97 | BenF_6_-Ben | -6.12 | -5.36 | 1.00 | 3505.44 | 96.00 | 0.76 | 0.00 |
| 98 | Chloromethane-Formaldehyde | -1.33 | -0.78 | 1.00 | 1830.98 | 26.00 | 0.39 | -0.16 |
| 99 | Bromomethane-Formaldehyde^b^ | -1.90 | -0.90 | 1.00 | 764.79 | 26.00 | 0.83 | -0.18 |
| 100 | Iodomethane-Formaldehyde^b^ | -2.26 | -1.45 | 1.00 | 860.50 | 26.00 | 0.93 | 0.12 |
| 101 | F_3_chloromethane-Formaldehyde | -2.22 | -1.57 | 1.00 | 1337.34 | 44.00 | 0.68 | 0.03 |
| 102 | F_3_bromomethane-Formaldehyde^b^ | -2.51 | -2.10 | 1.00 | 1438.31 | 44.00 | 1.00 | 0.59 |
| 103 | F_3_iodomethane-Formaldehyde | -3.96 | -3.31 | 1.00 | 1596.99 | 44.00 | 0.77 | 0.12 |
| 104 | BenCl-Acetone | -1.73 | -0.85 | 1.00 | 3943.81 | 60.00 | 0.64 | -0.24 |
| 105 | BenBr-Acetone | -2.61 | -2.74 | 1.00 | 4198.42 | 60.00 | -0.32 | -0.19 |
| 106 | BenI-Acetone^b^ | -2.89 | -3.79 | 1.00 | 4384.11 | 60.00 | -0.33 | 0.57 |
| 107 | BenCl-NMe_3_^b^ | -2.92 | -1.60 | 1.00 | 3696.94 | 62.00 | 0.52 | -0.80 |
| 108 | BenBr- NMe_3_ | -3.41 | -2.34 | 1.00 | 3728.94 | 62.00 | 1.44 | 0.37 |
| 109 | BenI- NMe_3_ | -5.79 | -5.09 | 1.00 | 3905.20 | 62.00 | 0.71 | 0.02 |
| 110 | BenBr-MeSH^b^ | -2.15 | 0.08 | 1.00 | 3226.31 | 50.00 | 2.39 | 0.16 |
| 111 | BenI-MeSH^b^ | -1.65 | -0.81 | 1.00 | 3472.59 | 50.00 | 2.27 | 1.42 |
| 112 | CH_3_Br-Ben | -1.81 | -0.37 | 2.00 | 1580.42 | 44.00 | 1.45 | 0.00 |
| 113 | CH_3_I-Ben | -2.40 | -0.82 | 2.00 | 2943.12 | 44.00 | 1.67 | 0.08 |
| 114 | CF3Br-Ben | -3.03 | -1.32 | 2.00 | 2789.08 | 62.00 | 1.79 | 0.08 |
| 115 | CF_3_I-Ben^b^ | -3.01 | -2.02 | 2.00 | 3076.28 | 62.00 | 1.90 | 0.91 |
| 116 | TrifluorometOH-Water | -9.77 | -9.89 | 1.00 | 644.96 | 40.00 | -0.22 | -0.10 |
| 117 | TrichlorometOH-Water | -10.30 | -10.44 | 1.00 | 1141.53 | 40.00 | -0.03 | 0.10 |
| 118 | HF-MeOH | -9.59 | -11.55 | 1.00 | 879.03 | 22.00 | -1.96 | 0.00 |
| 119 | HF-MeNH_2_ | -14.32 | -18.90 | 1.00 | 288.12 | 32.00 | -4.58 | 0.00 |
| 120 | Methanol-Fluoromethane | -2.94 | -2.48 | 1.00 | 593.70 | 28.00 | 1.41 | 0.95 |
| 121 | Methanol-Chloromethane | -2.50 | -1.53 | 1.00 | 764.34 | 28.00 | 2.25 | 1.27 |

- ^a^ The errors regards to CCSD(T)/CBS benchmark NCI valules.
- ^b^The molecules in the test set.
