# Supplementary material for: A machine learning correction for DFT non-covalent interactions based on the S22, S66 and X40 benchmark databases
Source: J Cheminform. 2016 May 3;8:24. doi: 10.1186/s13321-016-0133-7 (PMC4855356; doi:10.1186/s13321-016-0133-7)
Supplement: Supplementary file 7 — 10.1186/s13321-016-0133-7 The NCI descriptors and errors based on WB97XD/6-31G*(vac) calculations. [file 13321_2016_133_MOESM7_ESM.doc]

Table S6. The NCI descriptors and errors ^a^ based on WB97XD/6-31G*(vac) calculations

| NO. | Name | GRNN | NCI | D | E_lumo+1_ | N_ve_ | Error | Error new |
| --- | --- | --- | --- | --- | --- | --- | --- | --- |
| **S66** |  |  |  |  |  |  |  |  |
| 1 | Water-MeOH^b^ | -4.81 | -7.97 | 1.92 | 0.20 | 22.00 | -2.27 | 0.89 |
| 2 | Water-MeNH_2_^b^ | -7.23 | -9.59 | 3.48 | 0.21 | 22.00 | -2.55 | -0.20 |
| 3 | Water-Peptide^b^ | -7.64 | -10.42 | 5.14 | 0.15 | 38.00 | -2.20 | 0.58 |
| 4 | MeOH dimer | -5.03 | -8.45 | 2.28 | 0.19 | 28.00 | -2.60 | 0.82 |
| 5 | MeOH-MeNH_2_^b^ | -7.98 | -10.55 | 3.82 | 0.20 | 28.00 | -2.88 | -0.31 |
| 6 | MeOH-Peptide | -7.84 | -11.28 | 5.24 | 0.15 | 44.00 | -2.95 | 0.50 |
| 7 | MeOH-Water | -4.94 | -7.85 | 2.41 | 0.22 | 22.00 | -2.76 | 0.15 |
| 8 | MeNH_2_-MeOH | -3.02 | -5.67 | 0.45 | 0.19 | 28.00 | -2.56 | 0.09 |
| 9 | MeNH_2_ dimer | -4.35 | -6.25 | 1.98 | 0.20 | 28.00 | -2.02 | -0.12 |
| 10 | MeNH_2_-Peptide | -5.23 | -7.65 | 2.96 | 0.15 | 44.00 | -2.17 | 0.25 |
| 11 | MeNH_2_-Water | -7.42 | -9.59 | 3.63 | 0.21 | 22.00 | -2.19 | -0.02 |
| 12 | Peptide-MeOH | -6.60 | -8.52 | 5.52 | 0.13 | 44.00 | -2.24 | -0.32 |
| 13 | Peptide-MeNH_2_ | -7.02 | -10.22 | 6.38 | 0.14 | 44.00 | -2.66 | 0.54 |
| 14 | Peptide dimer | -8.71 | -11.12 | 7.77 | 0.13 | 60.00 | -2.40 | 0.01 |
| 15 | Peptide-Water | -5.45 | -7.45 | 6.91 | 0.13 | 38.00 | -2.25 | -0.25 |
| 16 | Uracil dimer | -17.45 | -18.64 | 7.74 | 0.04 | 84.00 | -1.19 | 0.00 |
| 17 | Water-Pyridine | -6.28 | -8.91 | 4.31 | 0.05 | 38.00 | -1.94 | 0.70 |
| 18 | MeOH-Pyridine^b^ | -6.45 | -9.69 | 4.30 | 0.05 | 44.00 | -2.18 | 1.06 |
| 19 | AcOH dimer | -18.88 | -20.67 | 0.00 | 0.09 | 48.00 | -1.26 | 0.54 |
| 20 | AcNH_2_ dimer | -16.68 | -18.54 | 0.00 | 0.12 | 50.00 | -2.02 | -0.15 |
| 21 | AcOH-Uracil | -19.65 | -20.78 | 3.68 | 0.08 | 66.00 | -1.00 | 0.13 |
| 22 | AcNH2-Uracil^b^ | -19.78 | -20.82 | 5.37 | 0.09 | 66.00 | -1.35 | -0.32 |
| 23 | Pyr dimer | -3.19 | -5.35 | 2.81 | 0.05 | 60.00 | -1.55 | 0.61 |
| 24 | Ur dimer | -9.74 | -11.16 | 4.95 | 0.03 | 84.00 | -1.40 | 0.01 |
| 25 | Ben-Pyr | -3.10 | -4.77 | 2.17 | 0.07 | 60.00 | -1.43 | 0.24 |
| 26 | Ben-Ur | -4.97 | -7.15 | 4.04 | 0.07 | 72.00 | -1.55 | 0.63 |
| 27 | Pyr-Ur | -5.74 | -8.02 | 2.23 | 0.05 | 72.00 | -1.32 | 0.97 |
| 28 | Benzene-Ethene | -1.76 | -2.18 | 0.14 | 0.08 | 42.00 | -0.81 | -0.40 |
| 29 | Ur-Ethene^b^ | -3.95 | -4.36 | 4.39 | 0.08 | 54.00 | -1.03 | -0.62 |
| 30 | Ur-Ethyne | -3.92 | -4.26 | 4.31 | 0.09 | 54.00 | -0.56 | -0.23 |
| 31 | Pyr-Ethene^b^ | -2.32 | -2.86 | 2.26 | 0.06 | 42.00 | -1.06 | -0.52 |
| 32 | Pentane dimer | -2.90 | -5.96 | 0.00 | 0.16 | 64.00 | -2.20 | 0.86 |
| 33 | Neopen-Pentane | -2.42 | -4.11 | 0.06 | 0.18 | 64.00 | -1.51 | 0.18 |
| 34 | Neopen dimer | -2.24 | -2.86 | 0.00 | 0.17 | 64.00 | -1.10 | -0.48 |
| 35 | Cyclopen-Neopen | -2.48 | -4.13 | 0.01 | 0.17 | 62.00 | -1.73 | -0.08 |
| 36 | Cyclopen-Cyclopen | -2.58 | -4.66 | 0.00 | 0.17 | 60.00 | -1.68 | 0.41 |
| 37 | Ben-Cyclopen^b^ | -3.83 | -5.57 | 0.49 | 0.07 | 60.00 | -2.06 | -0.31 |
| 38 | Ben-Neopen^b^ | -3.49 | -4.37 | 0.56 | 0.07 | 62.00 | -1.52 | -0.65 |
| 39 | Ur-Pentane^b^ | -4.80 | -7.28 | 4.20 | 0.08 | 74.00 | -2.47 | 0.01 |
| 40 | Ur-Cyclopen | -4.33 | -6.16 | 4.34 | 0.08 | 74.00 | -2.07 | -0.24 |
| 41 | Ur-Neopen | -4.14 | -5.45 | 4.33 | 0.08 | 74.00 | -1.76 | -0.45 |
| 42 | Ethene-Pentane | -2.05 | -3.32 | 0.23 | 0.16 | 44.00 | -1.33 | -0.05 |
| 43 | Ethyne-Pentane^b^ | -1.88 | -2.82 | 0.22 | 0.12 | 44.00 | -1.10 | -0.16 |
| 44 | Peptide-Pentane | -3.96 | -6.53 | 3.46 | 0.14 | 62.00 | -2.27 | 0.30 |
| 45 | Ben dimer | -3.32 | -3.90 | 0.56 | 0.07 | 60.00 | -1.07 | -0.50 |
| 46 | Pyr dimer | -3.58 | -4.68 | 4.47 | 0.05 | 60.00 | -1.17 | -0.07 |
| 47 | Ben-Pyr | -2.90 | -4.42 | 2.84 | 0.06 | 60.00 | -1.13 | 0.40 |
| 48 | Ben-Ethyne | -2.10 | -3.69 | 0.51 | 0.07 | 40.00 | -0.83 | 0.75 |
| 49 | Ethyne dimer | -1.49 | -2.25 | 0.47 | 0.12 | 20.00 | -0.71 | 0.05 |
| 50 | Ben-AcOH | -4.07 | -6.37 | 1.57 | 0.06 | 54.00 | -1.64 | 0.65 |
| 51 | Ben-AcNH_2_ | -3.95 | -6.14 | 3.65 | 0.08 | 54.00 | -1.73 | 0.46 |
| 52 | Ben-Water | -3.15 | -5.03 | 2.68 | 0.06 | 38.00 | -1.75 | 0.13 |
| 53 | Ben-MeOH^b^ | -3.05 | -5.56 | 2.18 | 0.06 | 44.00 | -1.39 | 1.12 |
| 54 | Ben-MeNH_2_^b^ | -2.72 | -4.75 | 1.90 | 0.06 | 44.00 | -1.55 | 0.48 |
| 55 | Ben-Peptide | -4.08 | -7.26 | 4.25 | 0.06 | 60.00 | -2.01 | 1.17 |
| 56 | Pyr dimer | -3.73 | -5.21 | 0.01 | 0.06 | 60.00 | -0.97 | 0.50 |
| 57 | Ethyne-Water | -2.74 | -4.86 | 2.41 | 0.14 | 18.00 | -1.94 | 0.18 |
| 58 | Ethyne-AcOH | -4.72 | -6.62 | 1.56 | 0.11 | 34.00 | -1.65 | 0.25 |
| 59 | Pentane-AcOH | -2.90 | -4.93 | 1.55 | 0.14 | 56.00 | -2.02 | 0.00 |
| 60 | Pentane-AcNH_2_ | -3.85 | -5.94 | 3.39 | 0.14 | 56.00 | -2.41 | -0.32 |
| 61 | Ben-AcOH^b^ | -3.65 | -5.17 | 1.55 | 0.07 | 54.00 | -1.43 | 0.10 |
| 62 | peptide-Ethene^b^ | -2.56 | -4.46 | 3.54 | 0.11 | 42.00 | -1.46 | 0.44 |
| 63 | Pyr-Ethyne | -3.86 | -5.22 | 3.39 | 0.05 | 40.00 | -1.11 | 0.25 |
| 64 | MeNH^2^-Pyr^b^ | -3.27 | -6.34 | 2.00 | 0.06 | 44.00 | -2.38 | 0.70 |
| **S22** |  |  |  |  |  |  |  |  |
| 65 | Adenine-Thymine | -16.36 | -18.71 | 1.63 | 0.06 | 98.00 | -2.34 | 0.01 |
| 66 | Adenine-Thymine | -12.23 | -14.08 | 3.31 | 0.05 | 98.00 | -1.85 | 0.00 |
| 67 | Ammonia dimer | -2.74 | -5.14 | 0.00 | 0.20 | 16.00 | -1.97 | 0.43 |
| 68 | Water dimer | -4.84 | -7.67 | 1.91 | 0.21 | 16.00 | -2.65 | 0.18 |
| 69 | Methane dimer^b^ | -0.55 | -0.82 | 0.00 | 0.24 | 16.00 | -0.29 | -0.02 |
| 70 | Ethene dimer | -1.44 | -2.46 | 0.00 | 0.10 | 24.00 | -0.95 | 0.07 |
| 71 | Ethene-Ethyne^b^ | -1.56 | -2.40 | 0.49 | 0.14 | 22.00 | -0.87 | -0.03 |
| 72 | Formicacid dimer | -18.52 | -20.16 | 0.00 | 0.07 | 36.00 | -1.55 | 0.09 |
| 73 | Formamide dimer | -16.23 | -18.51 | 0.01 | 0.11 | 36.00 | -2.55 | -0.27 |
| 74 | Benzene-Ammonia | -2.52 | -3.72 | 2.29 | 0.06 | 39.00 | -1.37 | -0.17 |
| 75 | Methane-Benzene^b^ | -1.76 | -2.23 | 0.22 | 0.07 | 38.00 | -0.73 | -0.26 |
| 76 | Benzene dimer | -3.21 | -3.47 | 0.55 | 0.07 | 60.00 | -0.73 | -0.47 |
| 77 | Benzene dimer | -3.27 | -4.07 | 0.00 | 0.08 | 60.00 | -1.34 | -0.54 |
| 78 | Indole-Benzene | -5.54 | -7.44 | 2.49 | 0.06 | 74.00 | -1.71 | 0.19 |
| 79 | Indole-Benzene | -5.50 | -7.27 | 2.48 | 0.06 | 74.00 | -2.05 | -0.28 |
| 80 | Pyrazine dimer | -4.17 | -5.61 | 0.20 | 0.02 | 70.00 | -1.19 | 0.25 |
| 81 | 2-pyridoxine2-aminopyridine | -16.88 | -19.02 | 2.60 | 0.05 | 72.00 | -2.31 | -0.17 |
| 82 | Phenol dimer | -6.29 | -9.66 | 3.61 | 0.08 | 72.00 | -2.61 | 0.76 |
| 83 | Uracil dimer^b^ | -9.74 | -11.13 | 4.87 | 0.03 | 84.00 | -1.01 | 0.38 |
| 84 | Uracil dimer | -20.65 | -21.36 | 0.00 | 0.03 | 84.00 | -0.71 | 0.00 |
| 85 | Benzene-HCN | -4.09 | -5.66 | 3.63 | 0.05 | 40.00 | -1.20 | 0.37 |
| **X40** |  |  |  |  |  |  |  |  |
| 86 | Methane-F_2_ | -0.76 | -0.58 | 0.01 | 0.21 | 22.00 | -0.09 | -0.27 |
| 87 | Methane-Cl_2_ | -0.80 | -0.80 | 0.16 | 0.20 | 22.00 | 0.28 | 0.27 |
| 88 | Methane-Br_2_^b^ | -1.43 | -1.09 | 0.32 | 0.10 | 22.00 | 0.21 | -0.13 |
| 89 | Methane-I_2_ | -1.39 | -1.28 | 0.39 | 0.08 | 22.00 | 0.07 | -0.04 |
| 90 | Fluoromethane-Methane^b^ | -0.91 | -1.50 | 1.70 | 0.22 | 22.00 | -0.75 | -0.16 |
| 91 | Chloromethane-Methane | -1.03 | -1.26 | 2.05 | 0.17 | 22.00 | -0.28 | -0.05 |
| 92 | Trifluoromethane-Methane^b^ | -1.16 | -1.70 | 1.61 | 0.21 | 34.00 | -1.01 | -0.47 |
| 93 | Trichloromethane-Methane | -1.57 | -1.40 | 1.34 | 0.08 | 34.00 | -0.25 | -0.42 |
| 94 | Fluoromethane-Fluoromethane | -1.86 | -2.83 | 2.93 | 0.20 | 28.00 | -1.18 | -0.21 |
| 95 | Chloromethane-Chloromethane | -1.43 | -2.73 | 0.17 | 0.11 | 28.00 | -1.39 | -0.09 |
| 96 | BenF_3_-Ben | -4.42 | -6.12 | 0.28 | 0.07 | 78.00 | -1.71 | -0.01 |
| 97 | BenF_6_-Ben | -6.08 | -8.15 | 0.47 | 0.05 | 96.00 | -2.03 | 0.04 |
| 98 | Chloromethane-Formaldehyde | -1.18 | -1.29 | 3.34 | 0.12 | 26.00 | -0.12 | -0.01 |
| 99 | Bromomethane-Formaldehyde^b^ | -1.92 | -1.46 | 2.85 | 0.08 | 26.00 | 0.27 | -0.20 |
| 100 | Iodomethane-Formaldehyde | -2.39 | -2.19 | 2.58 | 0.05 | 26.00 | 0.19 | -0.01 |
| 101 | F_3_chloromethane-Formaldehyde | -2.15 | -2.32 | 2.44 | 0.09 | 44.00 | -0.07 | 0.09 |
| 102 | F_3_bromomethane-Formaldehyde | -2.70 | -3.04 | 2.85 | 0.06 | 44.00 | 0.07 | 0.40 |
| 103 | F_3_iodomethane-Formaldehyde^b^ | -3.80 | -4.04 | 3.47 | 0.03 | 44.00 | 0.04 | 0.28 |
| 104 | BenCl-Acetone | -2.66 | -3.54 | 2.65 | 0.06 | 60.00 | -2.05 | -1.17 |
| 105 | BenBr-Acetone^b^ | -2.70 | -3.69 | 2.65 | 0.06 | 60.00 | -1.26 | -0.27 |
| 106 | BenI-Acetone | -2.99 | -4.80 | 2.84 | 0.06 | 60.00 | -1.34 | 0.47 |
| 107 | BenCl-NMe_3_ | -3.09 | -2.56 | 0.83 | 0.07 | 62.00 | -0.44 | -0.98 |
| 108 | BenBr- NMe_3_^b^ | -3.24 | -3.41 | 0.08 | 0.07 | 62.00 | 0.36 | 0.54 |
| 109 | BenI- NMe_3_ | -4.01 | -5.67 | 0.84 | 0.07 | 62.00 | 0.13 | 1.79 |
| 110 | BenBr-MeSH | -2.29 | -0.39 | 2.89 | 0.06 | 50.00 | 1.93 | 0.03 |
| 111 | BenI-MeSH^b^ | -2.25 | -1.25 | 2.37 | 0.06 | 50.00 | 1.83 | 0.83 |
| 112 | CH_3_Br-Ben | -2.07 | -0.30 | 1.61 | 0.08 | 44.00 | 1.51 | -0.25 |
| 113 | CH_3_I-Ben | -2.18 | 0.75 | 1.25 | 0.07 | 44.00 | 3.23 | 0.30 |
| 114 | CF3Br-Ben^b^ | -2.99 | -2.09 | 0.95 | 0.07 | 62.00 | 1.02 | 0.12 |
| 115 | CF_3_I-Ben | -3.06 | -2.91 | 1.49 | 0.06 | 62.00 | 1.01 | 0.86 |
| 116 | TrifluorometOH-Water | -9.76 | -13.81 | 3.08 | 0.21 | 40.00 | -4.14 | -0.09 |
| 117 | TrichlorometOH-Water | -10.40 | -15.45 | 3.20 | 0.09 | 40.00 | -5.04 | 0.00 |
| 118 | HF-MeOH | -8.73 | -12.09 | 3.56 | 0.21 | 22.00 | -2.50 | 0.86 |
| 119 | HF-MeNH_2_ | -14.07 | -16.05 | 4.44 | 0.21 | 32.00 | -1.74 | 0.25 |
| 120 | Methanol-Fluoromethane | -4.57 | -6.83 | 2.27 | 0.20 | 28.00 | -2.94 | -0.67 |
| 121 | Methanol-Chloromethane | -3.55 | -5.58 | 1.29 | 0.16 | 28.00 | -1.80 | 0.23 |

- ^a^ The errors regards to CCSD(T)/CBS benchmark NCI valules.
- ^b^ The molecules in the test set.
