# Supplementary material for: A machine learning correction for DFT non-covalent interactions based on the S22, S66 and X40 benchmark databases
Source: J Cheminform. 2016 May 3;8:24. doi: 10.1186/s13321-016-0133-7 (PMC4855356; doi:10.1186/s13321-016-0133-7)
Supplement: Supplementary file 8 — 10.1186/s13321-016-0133-7 The NCI, descriptors and errors based on WB97XD6-31G* calculations. [file 13321_2016_133_MOESM8_ESM.docx]

Table S7. The NCI, descriptors and errors ^a^ based on WB97XD6-31G* calculations

| NO. | Name | GRNN | NCI | D | E_lumo+1_ | N_ve_ | Error | Error new |
| --- | --- | --- | --- | --- | --- | --- | --- | --- |
| **S66** |  |  |  |  |  |  |  |  |
| 1 | Water-MeOH^b^ | -5.05 | -6.43 | 3.31 | 0.21 | 22.00 | -0.73 | 0.65 |
| 2 | Water-MeNH_2_^b^ | -7.16 | -8.52 | 4.81 | 0.21 | 22.00 | -1.48 | -0.12 |
| 3 | Water-Peptide^b^ | -7.55 | -8.16 | 7.19 | 0.17 | 38.00 | 0.06 | 0.67 |
| 4 | MeOH dimer | -5.41 | -7.25 | 3.43 | 0.20 | 28.00 | -1.39 | 0.44 |
| 5 | MeOH-MeNH_2_^b^ | -8.08 | -9.86 | 4.65 | 0.21 | 28.00 | -2.20 | -0.41 |
| 6 | MeOH-Peptide | -7.78 | -9.17 | 7.12 | 0.16 | 44.00 | -0.83 | 0.56 |
| 7 | MeOH-Water | -5.32 | -6.87 | 3.64 | 0.21 | 22.00 | -1.78 | -0.23 |
| 8 | MeNH_2_-MeOH | -3.26 | -4.08 | 1.37 | 0.20 | 28.00 | -0.96 | -0.15 |
| 9 | MeNH_2_ dimer | -3.96 | -4.66 | 3.77 | 0.20 | 28.00 | -0.44 | 0.26 |
| 10 | MeNH_2_-Peptide | -5.16 | -5.20 | 4.43 | 0.16 | 44.00 | 0.28 | 0.32 |
| 11 | MeNH_2_-Water | -7.20 | -8.59 | 4.74 | 0.21 | 22.00 | -1.19 | 0.20 |
| 12 | Peptide-MeOH | -6.82 | -7.31 | 6.82 | 0.15 | 44.00 | -1.03 | -0.54 |
| 13 | Peptide-MeNH_2_ | -7.69 | -9.18 | 7.96 | 0.15 | 44.00 | -1.62 | -0.13 |
| 14 | Peptide dimer | -8.72 | -9.17 | 9.98 | 0.12 | 60.00 | -0.45 | 0.00 |
| 15 | Peptide-Water | -6.97 | -6.81 | 7.85 | 0.15 | 38.00 | -1.61 | -1.78 |
| 16 | Uracil dimer | -6.93 | -7.04 | 5.71 | 0.06 | 38.00 | -0.06 | 0.04 |
| 17 | Water-Pyridine | -17.45 | -13.20 | 9.99 | 0.03 | 84.00 | 4.24 | 0.00 |
| 18 | MeOH-Pyridine^b^ | -6.89 | -8.08 | 5.64 | 0.05 | 44.00 | -0.57 | 0.62 |
| 19 | AcOH dimer | -19.24 | -15.75 | 0.01 | 0.08 | 48.00 | 3.67 | 0.18 |
| 20 | AcNH_2_ dimer | -16.51 | -12.22 | 0.00 | 0.11 | 50.00 | 4.30 | 0.02 |
| 21 | AcOH-Uracil | -19.34 | -14.36 | 4.69 | 0.08 | 66.00 | 5.42 | 0.44 |
| 22 | AcNH2-Uracil^b^ | -19.68 | -13.34 | 6.74 | 0.08 | 66.00 | 6.13 | -0.21 |
| 23 | Pyr dimer | -3.42 | -4.41 | 3.84 | 0.05 | 60.00 | -0.61 | 0.38 |
| 24 | Ur dimer | -9.30 | -6.65 | 4.84 | 0.03 | 84.00 | 3.10 | 0.45 |
| 25 | Ben-Pyr | -3.33 | -3.81 | 2.93 | 0.06 | 60.00 | -0.47 | 0.01 |
| 26 | Ben-Ur | -5.11 | -5.96 | 5.64 | 0.07 | 72.00 | -0.37 | 0.48 |
| 27 | Pyr-Ur | -6.02 | -6.03 | 3.86 | 0.05 | 72.00 | 0.67 | 0.68 |
| 28 | Benzene-Ethene | -1.87 | -1.58 | 0.07 | 0.07 | 42.00 | -0.21 | -0.51 |
| 29 | Ur-Ethene^b^ | -4.30 | -3.58 | 5.76 | 0.08 | 54.00 | -0.25 | -0.97 |
| 30 | Ur-Ethyne | -4.21 | -2.89 | 5.72 | 0.08 | 54.00 | 0.80 | -0.52 |
| 31 | Pyr-Ethene^b^ | -2.69 | -2.32 | 2.94 | 0.06 | 42.00 | -0.51 | -0.88 |
| 32 | Pentane dimer | -3.23 | -5.84 | 0.00 | 0.16 | 64.00 | -2.08 | 0.53 |
| 33 | Neopen-Pentane | -2.54 | -3.94 | 0.08 | 0.18 | 64.00 | -1.33 | 0.07 |
| 34 | Neopen dimer | -2.27 | -2.78 | 0.00 | 0.17 | 64.00 | -1.02 | -0.50 |
| 35 | Cyclopen-Neopen | -2.60 | -3.98 | 0.02 | 0.17 | 62.00 | -1.58 | -0.20 |
| 36 | Cyclopen-Cyclopen | -2.73 | -4.48 | 0.01 | 0.17 | 60.00 | -1.49 | 0.25 |
| 37 | Ben-Cyclopen^b^ | -3.92 | -5.05 | 0.42 | 0.07 | 60.00 | -1.54 | -0.41 |
| 38 | Ben-Neopen^b^ | -3.48 | -4.01 | 0.44 | 0.07 | 62.00 | -1.16 | -0.63 |
| 39 | Ur-Pentane^b^ | -5.02 | -6.67 | 5.75 | 0.08 | 74.00 | -1.86 | -0.21 |
| 40 | Ur-Cyclopen | -4.84 | -5.76 | 5.80 | 0.08 | 74.00 | -1.67 | -0.75 |
| 41 | Ur-Neopen | -4.73 | -4.79 | 5.79 | 0.08 | 74.00 | -1.11 | -1.04 |
| 42 | Ethene-Pentane | -2.07 | -3.09 | 0.19 | 0.16 | 44.00 | -1.09 | -0.08 |
| 43 | Ethyne-Pentane^b^ | -2.23 | -2.39 | 0.23 | 0.12 | 44.00 | -0.67 | -0.51 |
| 44 | Peptide-Pentane | -4.00 | -5.65 | 4.78 | 0.15 | 62.00 | -1.40 | 0.26 |
| 45 | Ben dimer | -3.30 | -3.56 | 0.39 | 0.07 | 60.00 | -0.73 | -0.47 |
| 46 | Pyr dimer | -3.82 | -3.74 | 5.53 | 0.05 | 60.00 | -0.23 | -0.31 |
| 47 | Ben-Pyr | -3.24 | -3.73 | 3.34 | 0.06 | 60.00 | -0.43 | 0.05 |
| 48 | Ben-Ethyne | -2.35 | -2.99 | 0.41 | 0.07 | 40.00 | -0.13 | 0.51 |
| 49 | Ethyne dimer | -1.55 | -1.57 | 0.37 | 0.12 | 20.00 | -0.03 | -0.01 |
| 50 | Ben-AcOH | -3.87 | -4.40 | 2.14 | 0.06 | 54.00 | 0.32 | 0.86 |
| 51 | Ben-AcNH_2_ | -3.89 | -3.77 | 4.76 | 0.07 | 54.00 | 0.63 | 0.52 |
| 52 | Ben-Water | -2.93 | -3.65 | 2.86 | 0.06 | 38.00 | -0.36 | 0.36 |
| 53 | Ben-MeOH^b^ | -3.43 | -4.50 | 2.48 | 0.06 | 44.00 | -0.33 | 0.74 |
| 54 | Ben-MeNH_2_^b^ | -3.19 | -4.03 | 2.14 | 0.07 | 44.00 | -0.83 | 0.00 |
| 55 | Ben-Peptide | -4.91 | -6.04 | 5.27 | 0.06 | 60.00 | -0.79 | 0.34 |
| 56 | Pyr dimer | -3.51 | -3.22 | 0.01 | 0.05 | 60.00 | 1.01 | 0.73 |
| 57 | Ethyne-Water | -2.96 | -4.03 | 2.70 | 0.13 | 18.00 | -1.11 | -0.03 |
| 58 | Ethyne-AcOH | -4.44 | -3.91 | 1.95 | 0.11 | 34.00 | 1.06 | 0.53 |
| 59 | Pentane-AcOH | -2.91 | -4.44 | 2.09 | 0.15 | 56.00 | -1.53 | -0.01 |
| 60 | Pentane-AcNH_2_ | -3.98 | -5.11 | 4.72 | 0.15 | 56.00 | -1.58 | -0.45 |
| 61 | Ben-AcOH^b^ | -3.85 | -4.25 | 2.07 | 0.07 | 54.00 | -0.50 | -0.11 |
| 62 | peptide-Ethene^b^ | -3.77 | -3.64 | 4.92 | 0.10 | 42.00 | -0.63 | -0.77 |
| 63 | Pyr-Ethyne | -4.12 | -4.13 | 3.93 | 0.06 | 40.00 | -0.03 | -0.02 |
| 64 | MeNH^2^-Pyr^b^ | -3.91 | -4.21 | 3.76 | 0.06 | 44.00 | -0.25 | 0.05 |
| **S22** |  |  |  |  |  |  |  |  |
| 65 | Adenine-Thymine | -16.38 | -13.96 | 2.23 | 0.05 | 98.00 | 2.41 | -0.01 |
| 66 | Adenine-Thymine | -10.05 | -9.81 | 4.41 | 0.04 | 98.00 | 2.42 | 2.18 |
| 67 | Ammonia dimer | -2.08 | -2.18 | 0.00 | 0.20 | 16.00 | 0.99 | 1.09 |
| 68 | Water dimer | -5.06 | -6.22 | 3.32 | 0.21 | 16.00 | -1.20 | -0.04 |
| 69 | Methane dimer^b^ | -0.74 | -0.77 | 0.00 | 0.24 | 16.00 | -0.24 | -0.21 |
| 70 | Ethene dimer | -1.48 | -1.97 | 0.00 | 0.09 | 24.00 | -0.46 | 0.03 |
| 71 | Ethene-Ethyne^b^ | -1.61 | -1.81 | 0.40 | 0.13 | 22.00 | -0.28 | -0.08 |
| 72 | Formicacid dimer | -18.68 | -15.13 | 0.00 | 0.08 | 36.00 | 3.48 | -0.07 |
| 73 | Formamide dimer | -16.09 | -12.03 | 0.01 | 0.11 | 36.00 | 3.93 | -0.13 |
| 74 | Benzene-Ammonia | -2.50 | -2.91 | 2.45 | 0.07 | 39.00 | -0.56 | -0.15 |
| 75 | Methane-Benzene^b^ | -1.97 | -1.93 | 0.17 | 0.07 | 38.00 | -0.43 | -0.47 |
| 76 | Benzene dimer | -3.19 | -3.09 | 0.41 | 0.07 | 60.00 | -0.35 | -0.45 |
| 77 | Benzene dimer | -3.08 | -3.08 | 0.00 | 0.07 | 60.00 | -0.35 | -0.35 |
| 78 | Indole-Benzene | -5.57 | -5.46 | 3.48 | 0.06 | 74.00 | 0.27 | 0.16 |
| 79 | Indole-Benzene | -5.06 | -4.57 | 2.86 | 0.08 | 74.00 | 0.65 | 0.16 |
| 80 | Pyrazine dimer | -4.40 | -4.68 | 0.08 | 0.02 | 70.00 | -0.26 | 0.02 |
| 81 | 2-pyridoxine2-aminopyridine | -17.15 | -13.61 | 4.08 | 0.05 | 72.00 | 3.10 | -0.44 |
| 82 | Phenol dimer | -6.73 | -8.24 | 4.37 | 0.07 | 72.00 | -1.19 | 0.32 |
| 83 | Uracil dimer^b^ | -9.30 | -6.65 | 4.84 | 0.03 | 84.00 | 3.47 | 0.82 |
| 84 | Uracil dimer | -20.64 | -13.22 | 0.08 | 0.03 | 84.00 | 7.43 | 0.01 |
| 85 | Benzene-HCN | -4.14 | -3.89 | 4.10 | 0.06 | 40.00 | 0.57 | 0.32 |
| **X40** |  |  |  |  |  |  |  |  |
| 86 | Methane-F_2_ | -1.12 | -0.60 | 0.00 | 0.21 | 22.00 | -0.11 | -0.62 |
| 87 | Methane-Cl_2_ | -1.15 | -0.63 | 0.11 | 0.20 | 22.00 | 0.45 | -0.07 |
| 88 | Methane-Br_2_^b^ | -1.46 | -0.79 | 0.24 | 0.10 | 22.00 | 0.51 | -0.16 |
| 89 | Methane-I_2_ | -1.42 | -0.92 | 0.24 | 0.08 | 22.00 | 0.43 | -0.07 |
| 90 | Fluoromethane-Methane^b^ | -1.37 | -1.32 | 2.03 | 0.22 | 22.00 | -0.57 | -0.62 |
| 91 | Chloromethane-Methane | -1.26 | -1.09 | 2.57 | 0.17 | 22.00 | -0.11 | -0.28 |
| 92 | Trifluoromethane-Methane^b^ | -1.57 | -1.65 | 1.89 | 0.23 | 34.00 | -0.96 | -0.88 |
| 93 | Trichloromethane-Methane | -1.58 | -1.26 | 1.65 | 0.09 | 34.00 | -0.12 | -0.43 |
| 94 | Fluoromethane-Fluoromethane | -1.76 | -2.25 | 3.35 | 0.20 | 28.00 | -0.60 | -0.11 |
| 95 | Chloromethane-Chloromethane | -1.35 | -1.16 | 3.34 | 0.11 | 28.00 | 0.18 | -0.01 |
| 96 | BenF_3_-Ben | -4.43 | -5.30 | 0.16 | 0.06 | 78.00 | -0.90 | -0.03 |
| 97 | BenF_6_-Ben | -6.10 | -7.00 | 0.25 | 0.05 | 96.00 | -0.88 | 0.02 |
| 98 | Chloromethane-Formaldehyde | -1.27 | -1.16 | 3.92 | 0.11 | 26.00 | 0.01 | -0.10 |
| 99 | Bromomethane-Formaldehyde^b^ | -2.00 | -1.13 | 3.42 | 0.07 | 26.00 | 0.59 | -0.28 |
| 100 | Iodomethane-Formaldehyde | -2.38 | -1.62 | 2.93 | 0.05 | 26.00 | 0.76 | 0.00 |
| 101 | F_3_chloromethane-Formaldehyde | -2.33 | -1.85 | 2.99 | 0.09 | 44.00 | 0.40 | -0.09 |
| 102 | F_3_bromomethane-Formaldehyde | -3.20 | -2.46 | 3.45 | 0.05 | 44.00 | 0.64 | -0.10 |
| 103 | F_3_iodomethane-Formaldehyde^b^ | -4.02 | -3.41 | 4.31 | 0.03 | 44.00 | 0.67 | 0.06 |
| 104 | BenCl-Acetone | -3.22 | -3.62 | 3.55 | 0.06 | 60.00 | -2.13 | -1.73 |
| 105 | BenBr-Acetone^b^ | -3.22 | -3.45 | 3.12 | 0.06 | 60.00 | -1.02 | -0.80 |
| 106 | BenI-Acetone | -3.34 | -4.25 | 3.24 | 0.05 | 60.00 | -0.79 | 0.12 |
| 107 | BenCl-NMe_3_ | -3.15 | -2.17 | 1.34 | 0.06 | 62.00 | -0.06 | -1.03 |
| 108 | BenBr- NMe_3_^b^ | -3.22 | -2.77 | 0.27 | 0.06 | 62.00 | 1.01 | 0.56 |
| 109 | BenI- NMe_3_ | -4.25 | -5.04 | 0.92 | 0.06 | 62.00 | 0.77 | 1.56 |
| 110 | BenBr-MeSH | -2.35 | 0.15 | 3.64 | 0.06 | 50.00 | 2.47 | -0.03 |
| 111 | BenI-MeSH^b^ | -2.46 | -0.53 | 2.91 | 0.05 | 50.00 | 2.55 | 0.62 |
| 112 | CH_3_Br-Ben | -2.08 | -0.42 | 2.13 | 0.07 | 44.00 | 1.40 | -0.26 |
| 113 | CH_3_I-Ben | -2.10 | -1.00 | 1.79 | 0.07 | 44.00 | 1.48 | 0.39 |
| 114 | CF3Br-Ben^b^ | -3.03 | -1.63 | 0.86 | 0.07 | 62.00 | 1.48 | 0.08 |
| 115 | CF_3_I-Ben | -3.18 | -2.21 | 1.55 | 0.06 | 62.00 | 1.70 | 0.73 |
| 116 | TrifluorometOH-Water | -9.67 | -11.73 | 4.92 | 0.24 | 40.00 | -2.06 | 0.00 |
| 117 | TrichlorometOH-Water | -10.41 | -13.02 | 4.95 | 0.08 | 40.00 | -2.61 | 0.00 |
| 118 | HF-MeOH | -9.29 | -11.28 | 4.57 | 0.22 | 22.00 | -1.68 | 0.30 |
| 119 | HF-MeNH_2_ | -14.31 | -16.43 | 5.52 | 0.23 | **32.00** | -2.11 | 0.01 |
| 120 | Methanol-Fluoromethane | -4.36 | -5.29 | 3.20 | 0.21 | **28.00** | -1.40 | -0.47 |
| 121 | Methanol-Chloromethane | -3.51 | -3.34 | 1.64 | 0.16 | 28.00 | 0.44 | 0.27 |

- ^a^ The errors regards to CCSD(T)/CBS benchmark NCI valules.
- ^b^The molecules in the test set.
