# Supplementary material for: A machine learning correction for DFT non-covalent interactions based on the S22, S66 and X40 benchmark databases
Source: J Cheminform. 2016 May 3;8:24. doi: 10.1186/s13321-016-0133-7 (PMC4855356; doi:10.1186/s13321-016-0133-7)
Supplement: Supplementary file 12 — 10.1186/s13321-016-0133-7 The NCI, descriptors and errors based on PBE/6-31G* calculations. [file 13321_2016_133_MOESM12_ESM.docx]

Table S11. The NCI, descriptors and errors ^a^ based on PBE/6-31G* calculations

| NO. | Name | GRNN | NCI | D | E_lumo+1_ | N_ve_ | Error | Error new |
| --- | --- | --- | --- | --- | --- | --- | --- | --- |
| **S66** |  |  |  |  |  |  |  |  |
| 1 | Water-MeOH^b^ | -4.92 | -6.23 | 3.43 | 0.10 | 22.00 | -0.53 | 0.78 |
| 2 | Water-MeNH_2_^b^ | -7.39 | -8.38 | 4.97 | 0.10 | 22.00 | -1.34 | -0.35 |
| 3 | Water-Peptide^b^ | -7.41 | -7.51 | 7.33 | 0.06 | 38.00 | 0.71 | 0.81 |
| 4 | MeOH dimer | -5.12 | -6.90 | 3.58 | 0.09 | 28.00 | -1.05 | 0.73 |
| 5 | MeOH-MeNH_2_^b^ | -8.39 | -9.56 | 4.88 | 0.10 | 28.00 | -1.90 | -0.72 |
| 6 | MeOH-Peptide | -7.83 | -8.14 | 7.28 | 0.06 | 44.00 | 0.20 | 0.51 |
| 7 | MeOH-Water | -5.30 | -7.49 | 3.79 | 0.11 | 22.00 | -2.41 | -0.21 |
| 8 | MeNH_2_-MeOH | -2.19 | -3.20 | 1.69 | 0.09 | 28.00 | -0.08 | 0.92 |
| 9 | MeNH_2_ dimer | -3.74 | -3.43 | 4.09 | 0.10 | 28.00 | 0.80 | 0.48 |
| 10 | MeNH_2_-Peptide | -5.28 | -3.48 | 4.86 | 0.06 | 44.00 | 2.00 | 0.20 |
| 11 | MeNH_2_-Water | -7.68 | -8.45 | 5.04 | 0.10 | 22.00 | -1.04 | -0.28 |
| 12 | Peptide-MeOH | -6.45 | -5.79 | 7.12 | 0.04 | 44.00 | 0.49 | -0.17 |
| 13 | Peptide-MeNH_2_ | -7.12 | -7.40 | 8.40 | 0.05 | 44.00 | 0.16 | 0.44 |
| 14 | Peptide dimer | -7.29 | -6.34 | 10.63 | 0.00 | 60.00 | 2.38 | 1.43 |
| 15 | Peptide-Water | -6.23 | -6.25 | 8.04 | 0.04 | 38.00 | -1.05 | -1.03 |
| 16 | Uracil dimer | -17.45 | -10.78 | 10.23 | -0.07 | 84.00 | 6.67 | 0.00 |
| 17 | Water-Pyridine | -6.95 | -6.57 | 6.20 | -0.05 | 38.00 | 0.41 | 0.03 |
| 18 | MeOH-Pyridine^b^ | -6.96 | -7.32 | 6.17 | -0.05 | 44.00 | 0.19 | 0.55 |
| 19 | AcOH dimer | -19.24 | -14.75 | 0.01 | -0.03 | 48.00 | 4.66 | 0.17 |
| 20 | AcNH_2_ dimer | -16.20 | -10.98 | 0.01 | 0.00 | 50.00 | 5.54 | 0.33 |
| 21 | AcOH-Uracil | -19.28 | -12.41 | 4.59 | -0.03 | 66.00 | 7.38 | 0.51 |
| 22 | AcNH2-Uracil^b^ | -19.70 | -11.49 | 7.01 | -0.02 | 66.00 | 7.98 | -0.23 |
| 23 | Pyr dimer | -3.63 | -0.17 | 3.58 | -0.05 | 60.00 | 3.63 | 0.17 |
| 24 | Ur dimer | -9.18 | -0.22 | 4.37 | -0.06 | 84.00 | 9.53 | 0.57 |
| 25 | Ben-Pyr | -3.57 | 0.14 | 3.00 | -0.03 | 60.00 | 3.48 | -0.23 |
| 26 | Ben-Ur | -4.00 | -0.10 | 5.70 | -0.03 | 72.00 | 5.49 | 1.59 |
| 27 | Pyr-Ur | -5.70 | -0.37 | 2.83 | -0.05 | 72.00 | 6.33 | 1.00 |
| 28 | Benzene-Ethene | -1.81 | 0.82 | 0.01 | -0.03 | 42.00 | 2.19 | -0.45 |
| 29 | Ur-Ethene^b^ | -3.86 | -0.66 | 5.87 | -0.02 | 54.00 | 2.67 | -0.53 |
| 30 | Ur-Ethyne | -3.87 | -1.15 | 5.78 | -0.02 | 54.00 | 2.54 | -0.18 |
| 31 | Pyr-Ethene^b^ | -2.23 | 0.14 | 2.99 | -0.04 | 42.00 | 1.94 | -0.43 |
| 32 | Pentane dimer | -2.69 | -1.10 | 0.00 | 0.06 | 64.00 | 2.66 | 1.08 |
| 33 | Neopen-Pentane | -2.59 | -1.05 | 0.10 | 0.07 | 64.00 | 1.56 | 0.01 |
| 34 | Neopen dimer | -2.62 | -0.99 | 0.00 | 0.07 | 64.00 | 0.78 | -0.85 |
| 35 | Cyclopen-Neopen | -2.62 | -0.88 | 0.02 | 0.07 | 62.00 | 1.52 | -0.23 |
| 36 | Cyclopen-Cyclopen | -2.63 | -1.13 | 0.03 | 0.07 | 60.00 | 1.86 | 0.36 |
| 37 | Ben-Cyclopen^b^ | -2.92 | -0.89 | 0.39 | -0.03 | 60.00 | 2.62 | 0.59 |
| 38 | Ben-Neopen^b^ | -2.93 | -0.84 | 0.34 | -0.03 | 62.00 | 2.00 | -0.08 |
| 39 | Ur-Pentane^b^ | -3.95 | -1.49 | 5.99 | -0.03 | 74.00 | 3.32 | 0.86 |
| 40 | Ur-Cyclopen | -3.95 | -1.21 | 5.93 | -0.02 | 74.00 | 2.88 | 0.14 |
| 41 | Ur-Neopen | -3.95 | -1.04 | 5.91 | -0.02 | 74.00 | 2.65 | -0.26 |
| 42 | Ethene-Pentane | -2.17 | -0.69 | 0.11 | 0.04 | 44.00 | 1.30 | -0.17 |
| 43 | Ethyne-Pentane^b^ | -2.08 | -0.52 | 0.27 | 0.01 | 44.00 | 1.19 | -0.36 |
| 44 | Peptide-Pentane | -3.97 | -1.69 | 4.96 | 0.05 | 62.00 | 2.56 | 0.29 |
| 45 | Ben dimer | -2.91 | -0.75 | 0.28 | -0.03 | 60.00 | 2.08 | -0.08 |
| 46 | Pyr dimer | -3.69 | -1.20 | 5.80 | -0.05 | 60.00 | 2.30 | -0.18 |
| 47 | Ben-Pyr | -3.50 | -0.82 | 3.28 | -0.04 | 60.00 | 2.47 | -0.21 |
| 48 | Ben-Ethyne | -2.28 | -0.98 | 0.34 | -0.03 | 40.00 | 1.87 | 0.58 |
| 49 | Ethyne dimer | -1.48 | -0.96 | 0.38 | 0.01 | 20.00 | 0.58 | 0.06 |
| 50 | Ben-AcOH | -3.17 | -1.78 | 2.30 | -0.04 | 54.00 | 2.94 | 1.56 |
| 51 | Ben-AcNH_2_ | -3.77 | -1.76 | 4.85 | -0.03 | 54.00 | 2.65 | 0.63 |
| 52 | Ben-Water | -2.32 | -1.40 | 2.87 | -0.04 | 38.00 | 1.89 | 0.97 |
| 53 | Ben-MeOH^b^ | -2.45 | -1.83 | 2.38 | -0.04 | 44.00 | 2.34 | 1.72 |
| 54 | Ben-MeNH_2_^b^ | -2.35 | -1.08 | 2.11 | -0.03 | 44.00 | 2.12 | 0.85 |
| 55 | Ben-Peptide | -3.90 | -1.56 | 5.46 | -0.04 | 60.00 | 3.70 | 1.35 |
| 56 | Pyr dimer | -3.43 | -1.87 | 0.01 | -0.05 | 60.00 | 2.37 | 0.81 |
| 57 | Ethyne-Water | -2.89 | -4.30 | 2.80 | 0.02 | 18.00 | -1.38 | 0.04 |
| 58 | Ethyne-AcOH | -3.90 | -2.67 | 1.97 | 0.00 | 34.00 | 2.30 | 1.06 |
| 59 | Pentane-AcOH | -2.73 | -1.21 | 2.13 | 0.04 | 56.00 | 1.70 | 0.17 |
| 60 | Pentane-AcNH_2_ | -3.93 | -1.60 | 4.90 | 0.05 | 56.00 | 1.93 | -0.40 |
| 61 | Ben-AcOH^b^ | -3.01 | -0.66 | 2.08 | -0.03 | 54.00 | 3.09 | 0.74 |
| 62 | peptide-Ethene^b^ | -2.20 | -1.43 | 5.00 | 0.00 | 42.00 | 1.57 | 0.80 |
| 63 | Pyr-Ethyne | -3.62 | -3.39 | 4.05 | -0.04 | 40.00 | 0.71 | 0.49 |
| 64 | MeNH^2^-Pyr^b^ | -3.80 | -2.83 | 4.86 | -0.04 | 44.00 | 1.14 | 0.17 |
| **S22** |  |  |  |  |  |  |  |  |
| 65 | Adenine-Thymine | -16.38 | -11.48 | 2.19 | -0.05 | 98.00 | 4.89 | -0.01 |
| 66 | Adenine-Thymine | -12.22 | -3.48 | 6.85 | -0.05 | 98.00 | 8.75 | 0.01 |
| 67 | Ammonia dimer | -1.82 | -1.77 | 0.27 | 0.10 | 16.00 | 1.40 | 1.35 |
| 68 | Water dimer | -5.08 | -6.83 | 3.30 | 0.10 | 16.00 | -1.81 | -0.06 |
| 69 | Methane dimer^b^ | -0.59 | -0.24 | 0.02 | 0.13 | 16.00 | 0.29 | -0.06 |
| 70 | Ethene dimer | -1.53 | -0.68 | 0.00 | -0.02 | 24.00 | 0.83 | -0.02 |
| 71 | Ethene-Ethyne^b^ | -1.50 | -1.07 | 0.40 | 0.01 | 22.00 | 0.46 | 0.03 |
| 72 | Formicacid dimer | -18.63 | -14.13 | 0.00 | -0.04 | 36.00 | 4.48 | -0.02 |
| 73 | Formamide dimer | -16.01 | -10.94 | 0.01 | -0.01 | 36.00 | 5.02 | -0.05 |
| 74 | Benzene-Ammonia | -2.21 | -0.87 | 2.48 | -0.03 | 39.00 | 1.48 | 0.14 |
| 75 | Methane-Benzene^b^ | -2.03 | -0.28 | 0.12 | -0.03 | 38.00 | 1.22 | -0.53 |
| 76 | Benzene dimer | -2.90 | -0.66 | 0.27 | -0.03 | 60.00 | 2.08 | -0.16 |
| 77 | Benzene dimer | -2.77 | 0.42 | 0.01 | -0.02 | 60.00 | 3.15 | -0.04 |
| 78 | Indole-Benzene | -5.49 | -1.34 | 3.66 | -0.04 | 74.00 | 4.39 | 0.24 |
| 79 | Indole-Benzene | -5.31 | -0.11 | 3.14 | -0.02 | 74.00 | 5.11 | -0.09 |
| 80 | Pyrazine dimer | -4.34 | -0.66 | 0.09 | -0.08 | 70.00 | 3.76 | 0.08 |
| 81 | 2-pyridoxine2-aminopyridine | -17.22 | -11.17 | 3.72 | -0.05 | 72.00 | 5.54 | -0.51 |
| 82 | Phenol dimer | -6.58 | -5.58 | 4.47 | -0.03 | 72.00 | 1.47 | 0.47 |
| 83 | Uracil dimer^b^ | -9.30 | -0.21 | 4.58 | -0.06 | 84.00 | 9.91 | 0.82 |
| 84 | Uracil dimer | -20.64 | -10.51 | 0.01 | -0.07 | 84.00 | 10.14 | 0.01 |
| 85 | Benzene-HCN | -2.79 | -1.22 | 4.14 | -0.04 | 40.00 | 3.24 | 1.67 |
| **X40** |  |  |  |  |  |  |  |  |
| 86 | Methane-F_2_ | -1.26 | -1.33 | 0.26 | 0.10 | 22.00 | -0.84 | -0.77 |
| 87 | Methane-Cl_2_ | -1.28 | -0.59 | 0.32 | 0.10 | 22.00 | 0.49 | -0.20 |
| 88 | Methane-Br_2_^b^ | -1.65 | -1.04 | 0.77 | 0.02 | 22.00 | 0.26 | -0.35 |
| 89 | Methane-I_2_ | -1.56 | -0.81 | 0.64 | 0.02 | 22.00 | 0.54 | -0.22 |
| 90 | Fluoromethane-Methane^b^ | -1.27 | -0.91 | 2.00 | 0.11 | 22.00 | -0.16 | -0.52 |
| 91 | Chloromethane-Methane | -1.19 | -0.26 | 2.56 | 0.07 | 22.00 | 0.72 | -0.21 |
| 92 | Trifluoromethane-Methane^b^ | -1.26 | -1.48 | 1.83 | 0.12 | 34.00 | -0.79 | -0.56 |
| 93 | Trichloromethane-Methane | -1.92 | -0.26 | 1.69 | -0.03 | 34.00 | 0.89 | -0.77 |
| 94 | Fluoromethane-Fluoromethane | -2.47 | -1.93 | 3.53 | 0.08 | 28.00 | -0.29 | -0.82 |
| 95 | Chloromethane-Chloromethane | -1.32 | -0.22 | 4.95 | 0.00 | 28.00 | 1.12 | 0.02 |
| 96 | BenF_3_-Ben | -4.17 | -0.08 | 0.13 | -0.03 | 78.00 | 4.32 | 0.24 |
| 97 | BenF_6_-Ben | -6.08 | -1.23 | 0.24 | -0.04 | 96.00 | 4.89 | 0.04 |
| 98 | Chloromethane-Formaldehyde | -1.52 | -1.11 | 3.27 | 0.01 | 26.00 | 0.06 | -0.35 |
| 99 | Bromomethane-Formaldehyde^b^ | -2.36 | -2.22 | 3.06 | -0.04 | 26.00 | -0.50 | -0.64 |
| 100 | Iodomethane-Formaldehyde | -2.34 | -2.40 | 2.68 | -0.05 | 26.00 | -0.02 | 0.04 |
| 101 | F_3_chloromethane-Formaldehyde | -2.50 | -2.02 | 2.90 | -0.03 | 44.00 | 0.22 | -0.25 |
| 102 | F_3_bromomethane-Formaldehyde | -3.31 | -3.55 | 3.11 | -0.07 | 44.00 | -0.45 | -0.21 |
| 103 | F_3_iodomethane-Formaldehyde^b^ | -3.88 | -4.00 | 3.80 | -0.08 | 44.00 | 0.08 | 0.20 |
| 104 | BenCl-Acetone | -3.11 | -3.37 | 2.92 | -0.04 | 60.00 | -1.88 | -1.62 |
| 105 | BenBr-Acetone^b^ | -3.33 | -4.79 | 3.01 | -0.04 | 60.00 | -2.36 | -0.91 |
| 106 | BenI-Acetone | -3.37 | -5.19 | 3.23 | -0.04 | 60.00 | -1.73 | 0.09 |
| 107 | BenCl-NMe_3_ | -3.02 | -0.96 | 0.67 | -0.04 | 62.00 | 1.15 | -0.91 |
| 108 | BenBr- NMe_3_^b^ | -3.33 | -2.75 | 0.17 | -0.04 | 62.00 | 1.03 | 0.44 |
| 109 | BenI- NMe_3_ | -4.73 | -4.92 | 1.69 | -0.04 | 62.00 | 0.89 | 1.07 |
| 110 | BenBr-MeSH | -2.78 | -1.02 | 3.44 | -0.04 | 50.00 | 1.30 | -0.46 |
| 111 | BenI-MeSH^b^ | -2.81 | -1.77 | 2.68 | -0.04 | 50.00 | 1.31 | 0.27 |
| 112 | CH_3_Br-Ben | -2.33 | -0.89 | 2.40 | -0.03 | 44.00 | 0.92 | -0.52 |
| 113 | CH_3_I-Ben | -2.34 | -1.03 | 1.98 | -0.03 | 44.00 | 1.45 | 0.14 |
| 114 | CF3Br-Ben^b^ | -3.08 | -1.85 | 0.17 | -0.03 | 62.00 | 1.26 | 0.03 |
| 115 | CF_3_I-Ben | -3.20 | -1.94 | 0.85 | -0.03 | 62.00 | 1.97 | 0.71 |
| 116 | TrifluorometOH-Water | -9.67 | -12.04 | 5.20 | 0.12 | 40.00 | -2.37 | 0.00 |
| 117 | TrichlorometOH-Water | -10.41 | -14.49 | 5.88 | -0.03 | 40.00 | -4.09 | 0.00 |
| 118 | HF-MeOH | -9.20 | -9.83 | 5.32 | 0.11 | 22.00 | -0.24 | 0.40 |
| 119 | HF-MeNH_2_ | -14.31 | -17.47 | 5.88 | 0.12 | 32.00 | -3.15 | 0.00 |
| 120 | Methanol-Fluoromethane | -4.37 | -5.16 | 3.09 | 0.10 | 28.00 | -1.27 | -0.48 |
| 121 | Methanol-Chloromethane | -2.86 | -0.94 | 0.66 | 0.06 | 28.00 | 2.83 | 0.91 |

- ^a^ The errors regards to CCSD(T)/CBS benchmark NCI valules.
- ^b^The molecules in the test set.
